# Deep sequencing and de novo assembly of the mouse oocyte transcriptome define the contribution of transcription to the DNA methylation landscape

**DOI:** 10.1186/s13059-015-0769-z

**Published:** 2015-09-25

**Authors:** Lenka Veselovska, Sebastien A. Smallwood, Heba Saadeh, Kathleen R. Stewart, Felix Krueger, Stéphanie Maupetit-Méhouas, Philippe Arnaud, Shin-ichi Tomizawa, Simon Andrews, Gavin Kelsey

**Affiliations:** Epigenetics Programme, Babraham Institute, Cambridge, UK; Bioinformatics Group, Babraham Institute, Cambridge, UK; GReD, CNRS, INSERM, and Clermont University, 63001 Clermont-Ferrand, France; Department of Histology and Cell Biology, Yokohama City University School of Medicine, Yokohama, Japan; Centre for Trophoblast Research, University of Cambridge, Cambridge, UK

## Abstract

**Background:**

Previously, a role was demonstrated for transcription in the acquisition of DNA methylation at imprinted control regions in oocytes. Definition of the oocyte DNA methylome by whole genome approaches revealed that the majority of methylated CpG islands are intragenic and gene bodies are hypermethylated. Yet, the mechanisms by which transcription regulates DNA methylation in oocytes remain unclear. Here, we systematically test the link between transcription and the methylome.

**Results:**

We perform deep RNA-Seq and de novo transcriptome assembly at different stages of mouse oogenesis. This reveals thousands of novel non-annotated genes, as well as alternative promoters, for approximately 10 % of reference genes expressed in oocytes. In addition, a large fraction of novel promoters coincide with MaLR and ERVK transposable elements. Integration with our transcriptome assembly reveals that transcription correlates accurately with DNA methylation and accounts for approximately 85–90 % of the methylome. We generate a mouse model in which transcription across the *Zac1/Plagl1* locus is abrogated in oocytes, resulting in failure of DNA methylation establishment at all CpGs of this locus. ChIP analysis in oocytes reveals H3K4me2 enrichment at the *Zac1* imprinted control region when transcription is ablated, establishing a connection between transcription and chromatin remodeling at CpG islands by histone demethylases.

**Conclusions:**

By precisely defining the mouse oocyte transcriptome, this work not only highlights transcription as a cornerstone of DNA methylation establishment in female germ cells, but also provides an important resource for developmental biology research.

**Electronic supplementary material:**

The online version of this article (doi:10.1186/s13059-015-0769-z) contains supplementary material, which is available to authorized users.

## Background

The DNA methylome is particularly dynamic during germ cell specification and gametogenesis, relating to the epigenetic reprogramming required to ensure the renewal of totipotency at each generation [1, 2]. DNA methylation (DNAme) is globally erased during migration of primordial germ cells (PGCs) towards the genital ridge, followed by de novo establishment of new methylation landscapes that are different between male and female germlines. In females, DNAme is established after birth, during follicular growth, in oocytes in meiotic arrest. Of particular interest, DNAme occurs at a subset of CpG islands (CGIs) termed imprinted germline differentially methylated regions (igDMRs); this gamete-derived methylation is maintained allele-specifically after fertilisation and acts as the basis for regulating genomic imprinting and its hundred mono-allelically expressed genes [3]. The recent development of methods combining bisulfite conversion as a means to determine methylation levels and high-throughput sequencing for low amounts of starting material have allowed the detailed profiling of the DNAme landscapes of germ cells and pre-implantation embryos [4–7]. Notably, these studies have revealed that whilst many CGIs are methylated in oocytes, most are not related directly to genomic imprinting but, nevertheless, a significant amount of oocyte-derived DNAme is present in embryonic day (E)3.5 pre-implantation blastocysts [5, 8, 9]. Yet, aside from this descriptive information, the mechanisms by which DNAme is established and regulated in oocytes, and its biological function aside from genomic imprinting, are still largely unclear.

Acquisition of DNAme at a genomic locus is likely to require integration of a combination of several factors, such as DNA sequence, specific *trans*-acting factors, and cross-talk between histone modifications and DNA methyltransferases (DNMTs) [2, 10]. Focusing on the *Gnas* imprinted locus, we established a functional link between transcription across an igDMR from an upstream transcription start site (TSS) and establishment of DNAme during oogenesis [11]. Similar results were subsequently obtained by others, as well as for the *Snrpn* imprinted locus [12, 13]. In addition, by performing reduced representation bisulfite sequencing (RRBS) in mature oocytes, we found that methylated CGIs are preferentially located within transcription units, highlighting a potential global role for transcription in determining the DNAme landscape of female germ cells [5]. This conclusion later received support when the first whole genome DNA methylome of these cells was reported, with evidence that gene bodies were enriched in DNAme [4]. The mechanistic role for transcription in DNAme establishment is likely (at least in part) to be a consequence of how the targeting of DNMT3A, and its co-factor DNMT3L, is regulated by histone post-translational modifications. Indeed, while histone 3 lysine 4 (H3K4) methylation has been shown to inhibit interaction of DNMT3A and DNMT3L with nucleosomes, H3K36me3 (a transcription elongation mark) enhances DNMT3A activity [14, 15]. These properties of the de novo methylation complex suggest that transcription could account for the majority of the oocyte methylome. Yet to what extent transcription controls DNAme establishment is undetermined and represents an unresolved question towards a full understanding of epigenetic reprogramming during development.

Regulation of transcription in oocytes is unique because of the distinctive nature and biological roles of these cells. They are highly transcriptionally active prior to and during the establishment of DNAme — with abundant accumulation of transcripts — and transcriptionally silent when mature. These transcripts serve not only to control oogenesis but also as a “maternal pool” for the regulation of pre-implantation development until zygotic and mid-preimplantation embryonic gene activation [16]. While our knowledge of the mouse oocyte transcriptome has greatly improved in recent years due to the development of RNA sequencing (RNA-Seq) for low amounts of input [4, 5, 16–18], such studies are limited because they relied on the annotated reference genome as a basis for their analysis, leading de facto to a loss of potentially critical information. Indeed, we have, for example, revealed that expression of imprinted genes in oocytes can be controlled by non-annotated oocyte-specific TSSs, and multiple studies in pluripotent and somatic cells have revealed the existence of non-coding RNAs (ncRNAs) which are not indexed in reference annotations [11, 19]. Therefore, to properly evaluate the contribution that transcription makes to patterning the oocyte methylome, a comprehensive description of the oocyte transcriptome and promoter use is required.

In this study, we set out to define precisely the correlation between transcription and the DNAme landscape in the following integrated approach. We sought to generate a high-quality transcriptome annotation by deep RNA-Seq of oocytes during follicular growth at the time of active de novo DNAme, with a particular focus on the identification and characterization of novel genes and TSSs; this analysis revealed a key role for transposable element (TE) expression in determining oocyte-specific transcription events. From nucleotide-resolution maps, we analysed the distribution of DNAme in the oocyte, and determined that the genome is partitioned into large-scale hypermethylated and hypomethylated domains, a distinctive feature of the oocyte methylome. By integrating these datasets, we assessed the coincidence of transcription units with hypermethylated domains. By this analysis, transcription accounts for up to 90 % of the methylome, but there are also exceptions to a simple, transcription-dependent model. Finally, we functionally demonstrated the requirement of transcription in establishing DNAme at all CpGs of a locus using transgenic mice.

## Results and discussion

### Our deep RNA sequencing approach outclasses previously published datasets

Several limitations were present in the datasets published by us and others prior to and during the course of this project, irrespective of their overall low sequencing depth [4, 5, 16–18, 20, 21]. First, apart from one study [16], only the poly-adenylated (poly(A))-enriched fraction was sequenced, while much evidence demonstrates the existence of long non-poly(A) transcripts transcribed by RNA polymerase II in mammalian cells [22]. Second, these data were mostly not strand-specific (i.e., there was no information on transcription orientation), hence limiting the accurate identification of alternative TSSs, for example. Finally, the datasets were generated from transcriptionally silent fully grown germinal vesicle and metaphase II oocytes, after DNAme establishment, and therefore potentially lacked transcripts expressed during early oocyte growth but degraded before the completion of oocyte development.

**Fig. 1:**
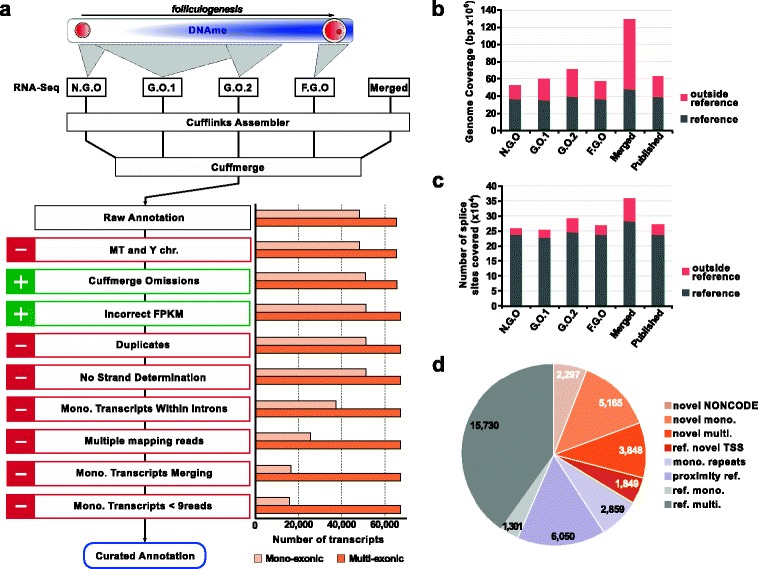
Oocyte transcriptome assembly. **a** Overview of the strategy used for the oocyte transcriptome assembly, with the different oocyte stages sequenced in relation to DNAme establishment (*top*), the curations made to the raw Cufflinks annotation (*bottom left*) and the corresponding changes in transcript numbers (*bottom right*). **b** Fraction of the genome covered by at least five non-redundant reads in our datasets, our merged datasets (*Merged*) and the merged published oocyte RNA-Seq datasets (*Published*; Table S2 in Additional file 2). **c** Number of reference splice sites covered by at least five non-redundant reads in our datasets, our merged datasets (*Merged*) and the merged published oocyte RNA-Seq datasets (*Published*). **d** Composition of the oocyte transcriptome: *novel NONCODE* corresponding to non-reference transcripts present in the NONCODEv4 database (±5 kbp); *ref. novel TSS* corresponding to reference transcripts for which an upstream TSS active in oocytes has been identified; *mono. repeats* corresponding to mono-exonic transcripts matching expressed TEs; *proximity ref.* corresponding to transcripts within 1 kbp or 5 kbp of reference genes for multi-exonic and mono-exonic transcripts, respectively. *FPKM* fragments per kilobase of transcript per million mapped reads

To circumvent these limitations, we generated strand-specific RNA-Seq libraries using ribosomal RNA depletion on oocytes isolated at different stages of follicular growth (i.e., non-growing oocytes (NGOs); growing oocytes (GOs; GO1 for mice aged 8–14 days post-partum (dpp), GO2 for mice aged 15 dpp); fully grown oocytes (FGOs)) (Table 1; Fig. 1a). Libraries were sequenced with 100 base pair (bp) paired-end reads, with a total number of reads generated of ~280 million, of which ~190 million were concordant paired-end reads. This resulted in a total of 129.7 Mbp covered by at least five unique reads, 80.7 Mbp of which were located outside the reference genome annotation (merging of Ensembl, University of California, Santa Cruz (UCSC) and RefSeq non-redundant transcripts isoforms). This represented an increase of 203.5 % over all the previously published datasets merged together (63.7 Mbp, 74.0 Mbp outside reference) (Fig. 1b; Figure S1a in Additional file 1). In addition, we reliably identified (covered by at least five unique reads) 283,171 splice junctions/exon boundaries matching the reference annotation and 74,037 novel ones, representing again a significant increase over the published datasets (258,033 and 33,782, respectively) (Fig. 1c; Figure S1a in Additional file 1).

**Table 1:** RNA-Seq samples and sequencing characteristics

Sample ID	Type	Age of mice (dpp)	Oocyte size	Number of oocytes	Raw sequencing reads	Uniquely mapped reads
NGO	Non-growing oocytes	5	10–40 μm	1545	44,111,934	31,173,658
GO1	Growing oocytes	8–14	25–70 μm	1990	67,882,721	42,709,253
GO2	Growing oocytes	15	50–70 μm	1510	118,463,451	82,037,819
FGO	Fully grown oocytes	20	>70 μm	832	47,855,997	35,107,487
			Total	5877	278,314,103	191,028,217

### Definition of the mouse oocyte transcriptome

To generate our oocyte reference annotation, we used Cufflinks, a genome-guided transcriptome assembler, using a combination of strategies [23, 24]. We performed Cufflinks on all datasets individually or merged, in default genome-guided mode or in reference annotation-based transcript (RABT) mode [25]; this combination of strategies was used because it was found that the different options tested gave different results, necessitating a composite approach for accurate assembly. Non-transcribed reference annotated transcripts included in the RABT mode (53–64 % total transcripts) were removed based on their expression values (Figure S1b in Additional file 1). All different assembly annotations were then merged into a single oocyte transcriptome annotation using the program Cuffmerge (Fig. 1a). It is known that Cufflinks can generate artefacts [26], and the raw oocyte annotation had to be curated step-by-step to remove new transcripts for which true biological identity was contentious (Fig. 1a; Figure S1c in Additional file 1; “Materials and methods”).

The final oocyte transcriptome annotation consists of 82,939 transcripts forming 39,099 expressed genes (14,995 mono-exonic and 24,104 multi-exonic), where a gene is a transcription unit that may comprise multiple transcript isoforms. Out of these, 20,428 genes (52.3 %), representing 6877 multi- and 13,551 mono-exonic genes, were only found in our oocyte annotation, the rest corresponding to known or novel isoforms of transcripts present in the reference annotation (Fig. 1d; Figure S1d in Additional file 1; Table S1 in Additional file 2). However, some of these novel transcripts may still correspond to known ncRNAs not present in the reference, as well as incomplete annotations of extended known transcripts. Therefore, for higher confidence in the identification of genuinely novel genes, we excluded all genes overlapping or in close proximity, on the same strand, to reference genes or known ncRNAs from the NONCODEv4 database (±1 kbp and ±5 kbp for multi- and mono-exonic genes, respectively) [27]. Furthermore, mono-exonic genes representing expressed independent repetitive elements annotated in RepeatMasker were excluded. This strategy resulted in the higher confidence identification of 3848 novel multi-exonic genes and 5165 novel mono-exonic genes (23.1 % of total; 13,809 transcripts; Fig. 1d). It should be noted that using these empirical criteria could have led to the removal of true biological transcripts, and some artefacts may remain in our final annotation. We have tested multiple parameters of analysis, and we believe the approach presented here was the most stringent possible and is fully adequate for the characterization and analysis performed below.

### A fraction of novel oocyte transcripts are potentially coding

To validate our experimental approach, we examined how many novel oocyte transcripts defined by our transcriptome assembly could retrospectively be identified using the previously published oocyte datasets. We observed that 94.3 % of novel multi-exonic and 55.1 % of novel mono-exonic genes are detected in these datasets merged together (FPKM (fragments per kilobase of transcript per million mapped reads) > 0.008, defined using the same approach as in Figure S1b in Additional file 1), and logically the overlap is greater for more highly expressed genes (Figure S2a in Additional file 1). We also validated by RT-PCR a random selection of novel genes (14) with a 100 % success rate for both multi- and mono-exonic genes (Figure S2b in Additional file 1).

**Fig. 2:**
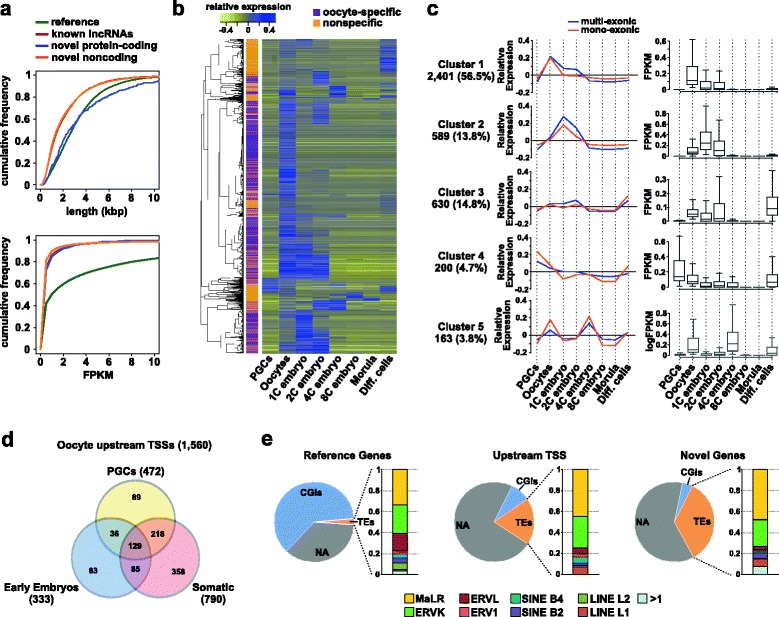
Characteristics of the novel oocyte genes identified. **a** Cumulative distributions of length and FPKM values of oocyte transcripts matching the reference annotation, known long ncRNAs (*lncRNAs*), and novel transcripts with and without protein-coding potential. **b** Hierarchical clustering of novel oocyte genes according to their relative expression (mean centred, log transformed FPKM, merged datasets) in oocytes versus PGCs, pre-implantation embryos, embryonic stemm cells, mouse embryonic fibroblasts and adult somatic tissues (*Diff. cells*) (see Table S2 in Additional file 2 for the full list of datasets). **c** Relative (*left*) and absolute (*right*) expression levels of novel oocyte genes in the largest clusters identified. The number of genes and corresponding percentages are indicated under each cluster. Expression values are log transformed FPKM. **d** Pie charts representing the proportion of TSSs overlapping CGIs, TEs or neither (*NA*) for reference genes, novel upstream TSSs of reference genes and novel genes. For each category, the proportion of each TE family is displayed as a bar graph. **e** Venn diagram representing the numbers of upstream TSSs of reference genes identified in our transcriptome assembly, in PGCs, early embryos and somatic tissues

While novel genes represent 23.1 % of all expressed genes in our oocyte transcriptome, they are, on average, shorter than reference genes (median of 2.5 kbp and 19.1 kbp, respectively) and represent only 7.6 % of the genomic fraction occupied by all expressed genes. In addition, the expression level of reference genes is substantially higher than that of novel genes (median FPKM of 2.65 and 0.19, respectively, from GO2 oocytes; Fig. 2a).

It is legitimate to assess what proportion of the novel oocyte genes is likely to have biological function. Therefore, we tested the potential of novel transcripts to encode proteins through the use of the specialised programs Coding Potential Calculator (CPC) and Coding-Non-Coding Index (CNCI) [28, 29]. CPC identified 841 mono- and 834 multi-exonic genes (18.6 % of all novel genes) and CNCI 100 mono- and 188 multi-exonic genes (3.2 %) as having coding potential. Even if predictions based on such bioinformatic tools must be considered with care (the overlap between CPC and CNCI is small — 171 genes), this suggests that we have identified a substantial number of ncRNAs. Interestingly, novel genes that appear to be oocyte-specific as they are not detected beyond the four-cell stage (~56 %; see below and Fig. 2b, c) are more likely to be non-coding than the novel genes detected in other developmental stages or in somatic tissues (49 versus 122 genes with coding potential based on both CPC and CNCI, respectively). Focusing on the protein domains of these coding-potential genes using blastx tool hits to the Uniprot database [30], we observed that at least nine novel genes possess known protein domains. Of interest, these include the Hop1p, Rev7p, and MAD2 (HORMA) domain, a chromatin-binding domain found in proteins regulating meiotic chromosome behaviour and DNA repair during meiosis. Interestingly, known proteins with HORMA domains have been demonstrated to play key roles in oogenesis [31].

### Identification of novel oocyte transcripts specifically expressed in female germ cells

If transcription patterns the DNA methylome of the oocyte, it is interesting to assess how many such transcription events are unique to the oocyte. To determine the fraction of novel transcripts that are specifically expressed in oocytes, we investigated their expression profiles in PGCs, throughout pre-implantation embryonic development (zygote to morula), in embryonic stem cells (ESCs) and various somatic tissues using relevant publicly available datasets (Table S2 in Additional file 2). Since most of these datasets are not strand-specific, we only analysed novel transcripts that do not overlap with others (2221 multi-exonic and 3210 mono-exonic genes). We performed hierarchical clustering analysis for novel genes expressed in at least one developmental stage (FPKM ≥ 0.1; 2075 multi-exonic and 2188 mono-exonic genes; Fig. 2b, c; Figure S2c in Additional file 1) and we observed that ~56 % of novel genes were classified as potentially oocyte-specific (54.1 % of multi- and 58.1 % of mono-exonic genes; principally clusters 1 and 2; Fig. 2c) based on their expression being detected in oocytes and up to four-cell embryos only, in accordance with a recent study examining timing of degradation of maternally provided transcripts after fertilisation [16] (Fig. 2b, c). Focusing on the PGC:oocyte transition, we determined that only 13.2 % of novel genes appear to be expressed already in PGCs (principally cluster 4), suggesting a profound remodelling of the transcriptome during specification of oocytes. However, it should be noted that inaccuracies could potentially arise from comparing datasets generated by different methods, and we cannot exclude at this stage that some of the novel oocyte genes are expressed at low levels at other developmental stages but are not detected in the respective datasets analysed.

### Characterization of novel transcription start sites reveals the contribution of transposable elements to the oocyte transcriptome

Previous results from our laboratory highlighted, in the context of genomic imprinting, the existence of alternative TSSs in oocytes non-annotated in the genome reference [11]. To investigate this genome-wide, we focused on genes for which TSSs are located in separate novel exons and outside reference TSS-associated CGIs. Using these criteria, we identified new upstream promoters active in oocytes for 1849 multi-exonic reference genes (10.8 % total expressed; Fig. 1d). Of note, the median distance between the reference and novel TSS was 5.3 kbp. Similar to novel genes, 79.9 % of these novel TSSs can be retrospectively classified as expressed/active in published oocyte datasets, and RT-PCR assays confirmed the expression of nine out of twelve randomly selected novel TSSs (this incomplete success could be attributed to limitation in primer design and sensitivity of detection in material of limiting availability; Figure S3a, b in Additional file 1). Interestingly, novel upstream TSSs of reference genes are less often located within CGIs compared with reference-annotated TSSs (8.7 % versus 49.4 %, respectively). This is similar to all novel transcripts identified in our oocyte annotation, with only 4.6 % (410) having a CGI-associated TSS (62 % for CGI-associated TSSs of reference genes expressed in the oocytes) (Fig. 2d).

By measuring the activity of the novel upstream TSSs of reference genes in other developmental stages, we found that 35.7 % appear to be oocyte-specific, as they were not detected in PGCs, eight-cell embryos, morula or any of the other cell types examined (1560 analysed genes with TSSs not overlapping with other genes) (Fig. 2e; Figure S3f in Additional file 1). Importantly, only 30.3 % of all novel upstream TSSs were detected in PGCs, highlighting again the substantial remodelling of the transcriptome associated with oocyte specification. Classifying genes based on their expression from upstream or reference TSSs shows that the most common pattern is that the gene is expressed from the upstream TSS in oocytes, but from the reference TSS in PGCs, embryos and differentiated cells (Figure S3f in Additional file 1).

Next, we aimed to identify common features for the novel TSSs active in oocytes (novel transcripts plus alternative TSSs of reference genes). A particularity of oocytes is the high transcriptional activity of TEs, and it was reported that TEs could act as promoters for a limited number of transcripts in mouse oocytes and ESCs [17, 32, 33]. To investigate this further, we first quantified the expression of TEs in our oocyte datasets. This revealed that the ERVK and especially MaLR families from the long terminal repeat class are highly expressed, in accordance with previous observations [33, 34] (Figure S3c in Additional file 1). Importantly, we found that TE-associated TSSs are found in 34.6 % (3121) of novel genes, and in 20.4 % (377) of novel upstream TSSs of reference genes; this is significantly higher than for annotated TSSs of expressed reference genes (478; 2.5 %). However, and of particular interest, only MaLR and ERVK elements act as TSSs more often than expected by chance based on occupancy of intergenic regions by individual TE families, with 282 novel upstream TSSs of reference genes and 2607 TSSs of novel genes coinciding with these TEs (Fig. 2d; Figure S2d in Additional file 1). Of note, the expression of novel genes with MaLR- and ERVK-associated TSSs (median FPKM values 0.259 and 0.325, respectively) is higher than novel genes with TSSs in unique sequences (median FPKM value 0.168, GO2 dataset) (Figure S3e in Additional file 1). In addition, ERVK and MaLR elements associated with promoters of novel genes are hypomethylated (18.3 % and 8.7 %, respectively) compared with the genome average (36.8 % and 33.4 %, respectively) and intergenic regions (28.0 % and 17.1 %, respectively).

### The oocyte DNA methylome is composed of large-scale hypermethylated and hypomethylated domains

**Fig. 3:**
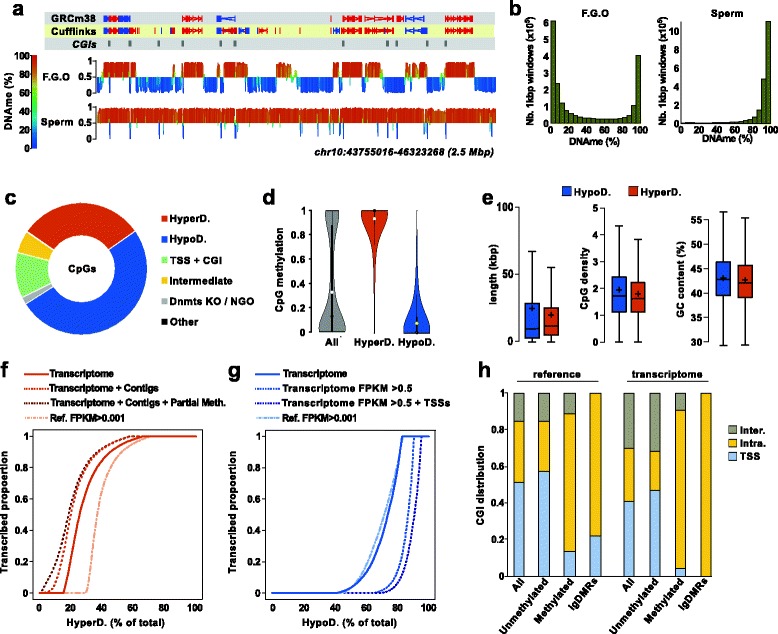
Oocyte methylome and correlation with transcriptome. **a** Visualization of the DNAme landscapes of FGOs and sperm using 2-kbp running genomic windows with a 1-kbp step. Quantification is absolute percentage of DNAme, with the x-axis set at 50 % methylation. **b** Distribution of 1-kbp genomic windows in FGOs and sperm according to their percentage of DNAme. **c** Distribution of genomic CpGs according to the following features: HyperDs and HypoDs, TSSs and CGIs, regions with intermediate methylation (25–75 %), regions with >50 % DNAme in DNMT knock-outs (*Dnmts KO*) and NGOs, and none of the above (*Other*). **d** Violin plot representation of DNAme of CpGs in FGOs in the entire genome (*All*) and in HyperDs and HypoDs (*open circles* represent the mean, *dark circles* the median, and *black line* the 1.5 × interquartile range). **e** Boxplot representation of the distribution of length, CpG density and GC content within HyperDs and HypoDs (*lines* represent the median and *crosses* the mean). **f** HyperDs ordered according to their increasing overlap with transcription in oocytes, based on the expressed reference genes (*Ref. FPKM > 0.001*), our transcriptome assembly, our assembly combined with read contigs, our assembly/contig combined with transcribed regions of partial DNAme (>25 %) in DNMT KOs and NGOs. **g** HypoDs ordered according to their increasing overlap with transcription in oocytes, based on the expressed reference genes (*Ref. FPKM > 0.001*), our transcriptome assembly, our assembly excluding genes with FPKM ≤ 0.5 alone or including also alternative TSSs. **h** Genomic location of CGIs and igDMRs in relation to expressed genes in the reference annotation and our oocyte transcriptome assembly

Previous studies based on whole-genome bisulfite sequencing revealed that the global DNAme level in fully grown germinal vesicle oocytes is approximately 40 % [4, 35], with a strongly bimodal distribution of methylation of CpGs, in contrast to what is observed in sperm, ESCs and typical somatic tissues. By examining in detail the oocyte DNA methylome, we observed that methylated and unmethylated CpGs are not distributed randomly throughout the genome. Instead, analysis of DNAme levels of consecutive 1 kbp genomic windows revealed that methylated CpGs tend to cluster together, such that the DNA methylome is composed of large-scale hypermethylated domains (HyperD) and hypomethylated domains (HypoD) (Fig. 3a, b).

To assess the extent of the DNA methylome that depends on transcription, we defined HyperDs and HypoDs bioinformatically. For this, as described in detail in the “Materials and methods” section, we determined the methylation level of genomic intervals corresponding to 50 consecutive CpGs (median size of 5.4 kbp), with overlapping steps of ten consecutive CpGs. We then merged consecutive windows with similar methylation levels, using cutoffs of 75–100 % and 0–25 % for HyperDs and HypoDs, respectively. For better correlation between transcription and DNAme (see below), we excluded from the domain definitions genomic regions corresponding to promoters and CGIs, as these features are likely to be unmethylated and would split one HyperD into several HyperDs but still potentially associated with one transcriptional unit (the correlation between transcription and CGI methylation is investigated separately below). We also excluded regions with 50 % or higher methylation in DNMT3A- or DNMT3L-deficient oocytes or early NGOs, as it was not possible to conclude how much of the ultimate DNAme in these regions could be a result of de novo events (Figure S4a, b in Additional file 1). Of note, all the analyses listed below were also performed using domains defined with genomic windows of ten consecutive CpGs with five consecutive CpG steps (median size 940 bp); the results being essentially the same, we describe results only for the former (50/10) conditions for clarity.

Our experimental approach resulted in the definition of 21,044 HyperDs and 25,165 HypoDs (46,209 domains in total; Additional file 4). Importantly, the majority of genomic CpGs are represented within HyperDs and HypoDs with 30.7 % and 50.7 % total CpGs, respectively, the remaining corresponding principally to promoters and CGIs (11.2 %) and regions with intermediate levels of methylation in FGOs (5.5 % total CpGs, 25–75 % methylation level) (Fig. 3c). As expected, 90.0 % of CpGs in HyperDs are methylated (≥75 % DNAme, average methylation 91.4 %) and 89.3 % of CpGs in HypoDs are unmethylated (≤25 % DNAme, average methylation 8.3 %), validating the accuracy of our approach and the concept of large-scale domains (Fig. 3d). HyperDs appeared smaller with an average size of 35.9 kbp (median 20.9 kbp) compared with 59.2 kbp (median 24.9 kbp) for HypoDs, reflecting the overall genome methylation (40 %) (Fig. 3e). Of note, the average CpG density was similar for HyperDs and HypoDs (1.8 and 1.7, respectively; Fig. 3e).

### Hypermethylated domains overlap with active transcription units

In order to test the strength of the association between transcription and DNAme, we quantified the proportion of each domain overlapped by transcription events. Based on our oocyte transcriptome annotation, 74.3 % of HyperDs are overlapped by transcripts for at least 50 % of their length (63.2 % for 80 % of their length; Fig. 3f). Since our oocyte transcriptome assembly was very stringent and the Cufflinks assembly could have missed some transcripts, we also defined transcribed units as regions with a minimum of three overlapping reads in at least one of our oocyte RNA-Seq datasets. Based on this, we found that 79.8 % of HyperDs are associated with transcription events (>50 % of the domain overlapped by a transcription unit; Fig. 3f). When considering the total length of all HyperDs together, 88.8 % overlaps with transcription units. Logically the correlation with transcription is dependent on the size of the HyperD, but interestingly the overlap with transcription units is higher for large domains (91.1 % of HyperDs >50 kbp are overlapped by transcription units for >50 % of the domain, which is 20.9 % of all HyperDs) compared with small domains (71.9 % of HyperDs <10 kbp — 22.2 % of all HyperDs), suggesting that some short domains in particular may require additional mechanisms for their DNAme establishment (Figure S5a, b in Additional file 1).

Despite the strong association with transcription, our analysis revealed that 2052 HyperDs (9.8 % of total) and some parts of HyperDs do not appear to be associated with transcription events (<5 % of their length overlaps transcribed regions). Of note, these apparently non-transcribed HyperDs are relatively short compared with all HyperDs, with an average size of 17.5 kbp (median 13.1 kbp). We set out to identify other features of these HyperDs that could contribute to DNAme establishment. We found that, compared with transcriptionally silent HypoDs, these HyperDs are enriched in ERVK elements, and also in intermediate levels of methylation (25–50 %) in NGOs or DNMT3A- and DNMT3L-deficient oocytes. However, these features represent only 1.7 % of the total length of all HyperDs. Nevertheless, when considered with transcription, this revealed that only 9.5 % of the combined length of HyperDs is unaccounted for (Figure S5b in Additional file 1). Evidently, it could be that our RNA-Seq strategy failed to capture some transcription events. This is difficult to assess, but seems less likely for highly transcribed regions, and lowly transcribed regions are usually hypomethylated (see below). Another explanation for this could relate to DNAme spreading, as observed in different contexts such as TEs, but this remains controversial and has not been shown in a germ cell context [36]. Alternatively, a transcription-independent mechanism could exist, based possibly on the interaction of DNMT3A/3L with specific histone marks other than H3K36me3. Further development of ChIP-Seq protocols for low amounts of starting material would be necessary to investigate this.

Having found a significant proportion of novel transcripts identified by our deep RNA-Seq approach, we investigated in more detail how this class contributes to the DNAme landscape. Our oocyte transcriptome contains 83.0 % of the methylated CpGs versus 75.3 % for the reference annotation: 4.5 % of methylated CpGs are within new genes. Focusing on transcripts expressed from TEs as promoters, for both novel genes and alternative upstream TSSs, a direct association was found for 4.7 % of methylated CpGs in oocytes. Similarly, novel genes and TE-regulated transcripts account for 2.9 % and 5.7 % of methylated CGIs, respectively.

### Some expressed genes escape DNA methylation

If transcription were the predominant factor in determining DNAme in oocytes, it would be surprising to find active transcription units devoid of DNAme. Therefore, we investigated HypoDs for which our oocyte annotation revealed substantial overlap with transcription. These correspond to 26.2 % of all HypoDs (overlap of >50 % with transcription units), or 16.1 % of the total length of HypoDs, which is quite a significant proportion (Fig. 3g; Figure S5c in Additional file 1). Of note, transcribed HypoDs are relatively small (median length 9.4 kbp), with 51.9 % and 23.3 % of these domains shorter than 10 kbp and 5 kbp, respectively. This size consideration could explain why they escape de novo methylation since short genes typically have low enrichment in H3K36me3 irrespective of expression level [37].

We found that long transcribed HypoDs are frequently associated with genes with very low FPKM values and, in accordance with previous observations, we observed that gene body DNAme levels are positively correlated with transcription levels, likely reflecting degree of H3K36me3 enrichment [4]; indeed, genes with <0.5 FPKM are more often unmethylated than methylated, while the proportion of methylated genes increases with increasing FPKM value (Figure S5e in Additional file 1). We found that 46.2 % of transcribed HypoDs (median length 14.7 kbp, accounting for 11.0 % of the total length of HypoDs) are associated with genes with <0.5 FPKM. In addition, we found that some of the HypoDs defined (14.2 %; 972) correspond to alternative downstream promoters active in oocytes according to our transcriptome assembly; these are shorter on average (median length 4.4 kbp) and could be protected from de novo methylation by H3K4me2/me3 marks [15] (Fig. 3g; Figure S5c in Additional file 1). Taking into consideration our transcription-based model for de novo DNA methylation, 9.2 % of all HypoDs (3.7 % of total length of HypoDs) appear to be transcribed (>50 % overlap) but their methylation status is not directly explained (Figure S5d in Additional file 1).

This prompted us to investigate how many expressed genes escape DNAme. We first identified 318 genes with gene-body DNAme <25 %, but with characteristics of normally methylated genes (FPKM > 1 and at least 10 kbp in size). To examine this further, we generated contigs (at least three mapped reads) for each dataset and analysed the methylation level of each gene using a running window strategy. This approach was used to limit potential Cufflinks artefacts, where only a fraction of the wrongly annotated gene would actually be transcribed and methylated. This confirmed 52 large and highly expressed genes (41 genes present in the reference annotation) as unmethylated throughout their entire gene body, and therefore in contradiction to our transcription-based model (Table S3 in Additional file 2). Of note, these genes are expressed at high levels throughout folliculogenesis, prior to and after the onset of DNAme targeting. Although gene ontology analysis failed to report significant enrichment for the 41 reference genes, it nevertheless regrouped genes important for meiosis and germ cell development (*Sohlh2*, *Slit3*, *Syce1*, *Tes*), known transcription regulators (*Foxo6*, *Zbtb38*, and *Zfp219*), as well as members of the *Sox* and *Pax* families (*Sox13*, *Pax6*).

### Transcription and DNA methylation establishment at CGIs and igDMRs

Having demonstrated the substantial contribution of transcription to the global DNA methylome, we next focused on specific genomic features: CGIs. Our oocyte annotation redefined CGI location compared with the reference annotation, and these can be divided into four groups: 9439 CGIs associated with the most upstream TSS of the gene (41.0 % of total); 1666 CGIs intragenic but associated with downstream/alternative TSSs of the gene (7.2 % of total); 5043 CGIs intragenic and not overlapping a TSS (21.9 % of total); 6861 intergenic CGIs (29.8 % of total). Of relevance, and highlighting the benefits of our transcriptome assembly approach, we found that 18.6 % of intergenic CGIs according to the reference annotation are associated with genes in oocytes, and 13.6 % of CGIs originally classified as the most upstream TSS are found to be intragenic.

Based on whole-genome bisulfite sequencing data in FGOs, 2047 CGIs were found to be hypermethylated (≥75 %; 9.1 % total CGIs) and 19,547 hypomethylated (≤25 %; 87.1 % total CGIs). We found that 86.5 % (1771) of the methylated CGIs are located within transcription units, while 3.8 % (78) are associated with the most upstream TSS and 9.7 % (198) are intergenic (Fig. 3h). Of note, 47.9 % (135) of the methylated CGIs overlapping the most upstream TSSs in the reference annotation become intragenic in our oocyte transcriptome. This results either from the existence of alternative upstream TSSs, or from new overlapping transcripts that are in ~25 % of the cases transcribed in the antisense orientation and regulated by a promoter located downstream of the methylated CGI.

Looking in more detail into the exceptions to a transcription-based mode strictly based on our Cufflinks assembly, we found a large fraction of intergenic CGIs (48 %) were still overlapped by transcribed units defined as regions with at least three overlapping reads in at least one of the oocyte RNA-Seq datasets; this was the case for only 15.7 % of unmethylated intergenic CGIs (Chi-squared test, *p* value <0.0001). Similarly, we observed a tendency for DNAme to extend beyond the 3′ end of a gene (for the top 40 % of genes based on their expression, DNAme is still above 75 % at 1 kb downstream) and 18.7 % of methylated intergenic CGIs overlap with the first 1 kbp downstream of a gene. For the remaining TSSs and intergenic CGIs, we investigated their methylation level in NGOs, oocytes deficient in DNMT3A and DNMT3L, and sperm, but found less than ten to be methylated in these cases.

We next asked whether all CGIs located within transcription units acquire DNAme, as might be predicted from a transcription-based model. Out of the 2863 intragenic unmethylated CGIs, 41.5 % are in close proximity (within 2 kbp) of the most upstream TSS, or overlapping, or in close proximity to a “downstream” alternative TSS, which might preclude their de novo methylation on the basis of spread of H3K4 methylation. In addition, 41.5 % of intragenic unmethylated CGIs are embedded within larger hypomethylated domains, mostly located within weakly transcribed gene bodies that do not support DNAme establishment. Ultimately we found only 136 CGIs unmethylated but located within a highly transcribed unit and surrounded by a hypermethylated domain. In this case, their methylation state could relate to general mechanisms protecting against DNAme at these genomic elements, and their capability to adopt specific chromatin signatures solely based on their GC-rich sequence [38, 39]; further improvement in ChIP-Seq methodologies will allow this possibility to be investigated in more detail. In conclusion, we found that the transcriptome not only defines a large fraction of methylated CGIs, but could also account for the hypomethylated state of the majority of CGIs.

Having shown that transcription correlates with CGI methylation, we focused on the specific subclass of these genomic features: igDMRs. Based on the reference annotation, 5 out of 23 maternal igDMRs overlap promoter regions (*Peg10*, *Peg3*, *Slc38a4*, *AK008011*, and *Impact*), the remainder being within annotated transcription units. Our transcriptome assembly now allows us to identify novel upstream TSSs for the *Peg10*, *Peg3*, and *Impact* genes, and novel transcripts transcribing through the *AK008011* and *Slc38a4* igDMRs (Fig. 3h; Figure S6 in Additional file 1). A recent publication identified 11 new putative maternal igDMRs [7], and our transcriptome revealed an intragenic location for nine of them. For the remaining two, *AK086712* and *Pvt1*, the associated igDMRs appear to be intergenic according our transcriptome, but are nevertheless located with HyperDs. These results highlight that transcription is the only common feature of maternal igDMRs, to our knowledge, and could link oocyte-specific signalling pathways to the establishment of genomic imprinting.

### Transcription is functionally required for DNAme establishment at the *Zac1* locus

Using a mouse model we originally provided a functional demonstration of the importance of transcription in the establishment of DNAme at the igDMRs of the *Gnas* locus [11]. For technical reasons, however, the poly(A) cassette strategy we used to block transcription was not fully efficient, resulting in variable loss of methylation between mice and precluding the use of this model for more refined and mechanistic analysis. In addition the *Gnas* locus is particularly complex with multiple igDMRs controlling expression of multiple transcripts (including antisense). For these reasons, we decided to test in more detail the role of transcription in DNAme targeting at another, more tractable locus.

**Fig. 4:**
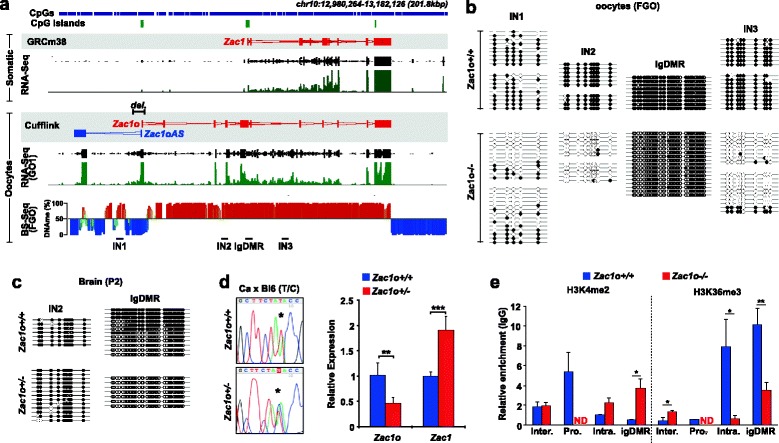
Transcription is required for DNAme targeting at the *Zac1* locus. **a** Visualization of the *Zac1* transcripts in somatic tissues (*top*) and in oocytes (*bottom*), as well as the DNAme landscape at this locus in FGOs. Deletion of *Zac1o* promoter is indicated by *del.* above the Cufflinks annotation, and below the DNAme profile are indicated the regions (*IN1*, *IN2*, *IN3*, *igDMR*) that are subsequently assessed for DNAme in (**b**, **c**). **b** DNAme status of *Zac1* igDMR and *Zac1o*/*Zac1oAS* intragenic regions in *Zac1o+/+* and *Zac1o−/−* FGOs. DNAme was assessed by bisulfite sequencing (BS-PCR) and each line represents an individual unique clone, with *open circles* representing unmethylated CpGs and *closed circles* methylated CpGs. **c** DNAme (BS-PCR) status of the *Zac1* igDMR and *Zac1o* IN2 intragenic region in *Zac1o+/+ and Zac1o+/−* neonatal (postnatal day 2 (*P2*)) brain. **d** Sequence traces (*left*) of RT-PCR products from neonatal brain from *Castaneus* crosses to *Zac1o+/+* and *Zac1o−/−*; the asterisk indicates the T/C single-nucleotide polymorphism. *Zac1o* and *Zac1* expression assessed by quantitative RT-PCR (*right*) in *Zac1o+/+* and *Zac1o+/−* neonatal brain (***p < 0.001, **p < 0.01, Student’s t-test). **e** ChIP-quantitative PCR quantification of H3K4me2 and H3K36me3 enrichment in growing oocytes (15 dpp) at *Zac1* igDMR, *Zac1o* intragenic regions and *Zac1o* intergenic regions (*ND* non-determined, *p < 0.05, **p < 0.01 Student’s t-test)

We decided to focus on the imprinted gene *Zac1* (*Plagl1*) principally because of the simplicity of the locus (only one imprinted gene, with igDMR overlapping the annotated canonical promoter), and because a human imprinted disorder is associated with *ZAC1* igDMR loss of methylation (transient neonatal diabetes mellitus) [40]. We previously identified by 5′ RACE (rapid amplification of 5′ complementary DNA ends) an oocyte alternative TSS, located ~30 kb upstream of the *Zac1* promoter (which is not active in oocytes), regulating the expression of a new *Zac1* transcript we named *Zac1o* [11]. Our transcriptome assembly validated the existence of *Zac1o*, and also revealed the presence of another, apparently non-coding transcript sharing the *Zac1o* CGI as promoter, but transcribed in the opposite direction, a transcript we named *Zac1oAS* (“AS” for antisense; Fig. 4a). Strikingly, a HyperD overlaps nicely with the oocyte *Zac1* transcription unit, which is particularly apparent at the 3′ end, where the HyperD and *Zac1o* transcription unit terminate at essentially the same genomic location (Fig. 4a). We generated a conditional knockout of the *Zac1o* promoter, resulting in the loss of expression of *Zac1o* and *Zac1oAS* in oocytes when crossed with the female germline specific CRE deleter transgenic line *Zp3-Cre* (Figure S7a, b in Additional file 1). As expected from the predictions of our transcription-based model, we found that DNAme fails to be established at the *Zac1* igDMR in the absence of transcription, and this loss of methylation is consistent across littermates and litters (Fig. 4b; Figure S7c in Additional file 1). Importantly, this was also the case for the majority of the gene body CpGs we tested, not just within the igDMR (Fig. 4b).

While the hypomethylated state of the *Zac1* igDMR is maintained after fertilisation and throughout development in embryos produced from *Zac1o*-deleted oocytes, CpGs located within the *Zac1o* gene body but outside the igDMR regained methylation, most likely following embryonic implantation [41] (Fig. 4c). Of interest, deletion in early embryos of the promoter regulating *Zac1o* transcription using *Sox2-Cre* mice did not affect methylation of the igDMR, in accordance with the nature of genomic imprinting, and revealing that transcription is not required for the maintenance of imprints (at least at *Zac1*) (Figure S7d in Additional file 1). Loss of methylation at the *Zac1* igDMR is functionally significant, since it is associated with twofold up-regulation and bi-allelic expression of *Zac1* transcripts in all tissues tested, assessed by crossing *Zac1o−/−* females with *Mus castaneus* males (Fig. 4d). To validate further this imprinting defect, we also examined the chromatin signature of the *Zac1* igDMR by performing ChIP analyses in *Zac1o+/+* and *Zac1o+/−* (maternal deletion in oocytes) embryos (Figure S7e in Additional file 1). This revealed that in embryos inheriting the *Zac1o* deletion from the oocyte, both parental alleles of the igDMR adopt a paternal epigenotype. Specifically, we noted a loss of the characteristic igDMR allele-specific histone modification signature: there was gain of H3K4me3 and H3K9ac on the maternal allele, marks normally enriched on the unmethylated paternal allele in *Zac1o+/+* embryos, and a marked decrease of the repressive H3K9me3 and H4K20me3, both enriched on the methylated maternal allele in *Zac1o+/+* embryos. This observation is reminiscent of those made in embryos 9.5 days post coitum derived from *Dnmt3L−/−* females that lack female germline-derived DNAme [42].

### Transcription is required for full chromatin remodelling at the *Zac1* igDMR

Using our *Zac1o* mouse model, we sought to investigate in more detail components of the mechanism linking transcription and DNAme. Aside from the role of H3K36me3 in promoting DNMT3A activity, transcription could be implicated in chromatin remodelling at CGIs, ensuring that protective marks are erased [2]. Importantly, the chromatin modifier H3K4me2 demethylase KDM1B has been implicated in DNAme establishment at the *Zac1* igDMR [43], and this prompted us to investigate a potential link between KDM1B and transcription. For this we optimised a ChIP-quantitative PCR assay based on a previously published micro-ChIP protocol [44]. We prepared chromatin from ~2000 growing oocytes (15 dpp) for each genotype (*Zac1o+/+* and *Zac1o−/−*), performed immunoprecipitation followed by whole genome amplification in duplicate with multiple quantitative PCR assays per genomic location (intergenic, intragenic, igDMR). To test the accuracy of our protocol, we investigated neutral loci expressed or not in oocytes (*Zp3*, *Ppia*, *Fam164b*; Figure S7f in Additional file 1). Focusing on the *Zac1* locus, and as mentioned above, in the absence of *Zac1o* transcription we found a significant decrease in H3K36me3 within the *Zac1o* gene body and igDMR. Importantly, we also found that H3K4me2 levels at the igDMR were significantly higher in *Zac1o−/−* oocytes versus *Zac1o+/+* oocytes (Fig. 4e). This result strongly suggests a connection between transcription and recruitment of KDM1B at the *Zac1* igDMR, and ultimately that transcription could be implicated in different levels of chromatin remodelling. Of relevance, it was recently reported that human KDM1B binding is enriched in active gene bodies, and it co-precipitates with elongating RNA polymerase II and other transcription elongation factors [45]. Development of reliable ChIP-Seq methods for low cell numbers will allow the connection between transcription and KDM1B at other igDMRs to be investigated in more detail; in addition, it will be important to address more widely the dependence on KDM1B of CGIs methylated in oocytes.

## Conclusions

Our work reveals that the real oocyte transcriptome is only approximated by the publicly available reference annotations. Indeed, we identified thousands of novel genes, coding or non-coding and, in particular, we discovered that many of these transcripts are linked to the de-repression and high expression of TEs from the MaLR and ERVK families in oocytes. Importantly, our transcriptome assembly can be used as a general resource for other scientists and developmental biology questions.

With this transcriptome assembly, we determined that transcription events could account for 85–90 % of DNAme established in the oocyte, including methylated CGIs and igDMRs; however, a small number of expressed genes escape DNAme, as well as a small number of CGIs within active transcription units. By establishing a tight genome-wide correlation between DNAme and active transcription units, as well as functionally demonstrating this at specific genomic loci, our work has wider implications. Indeed, it suggests that gene expression perturbations during oocyte follicular growth could result in alterations in DNAme in mature gametes, including at CGIs. Since a fraction of the oocyte DNA methylome is maintained to some extent in pre-implantation embryos just before the embryonic onset of de novo methylation (the biological consequence of this remains unclear), environmentally induced changes in gene expression in female germ cells could lead to alterations in the epigenome of the next generation, with possible transgenerational effects [5, 8].

It is difficult to precisely dissect the mechanisms by which transcription promotes DNAme establishment in oocytes due to the difficulty in obtaining large numbers of these cells. Nevertheless, in vitro biochemical evidence demonstrating a role for H3K36me3 in promoting DNMT3A catalytic activity is obviously central to our understanding, especially since DNMT3B is not active in oocytes. However, and surprisingly given our findings, recent work in mouse ESCs, derived neuronal progenitors, and the human colorectal carcinoma HCT116 cell line have showed that it is principally DNMT3B (and to a lesser extent DNMT3A) that is associated with gene-body methylation [46, 47]. Elucidating how DNMT3A specificity towards certain genomic features varies depending on the cellular context would be important to understand DNAme dynamics during early embryonic development and germ cell specification.

By revealing that H3K4me2 removal from the *Zac1* igDMR is impaired in the absence of transcription, our results suggest that the role of transcription in DNAme targeting is likely more complex than a simple interaction of DNMT3A with H3K36me3, and could involve the recruitment of histone remodellers or modifiers required for DNAme establishment. Transcription could also indirectly promote complete DNAme establishment by promoting nucleosome displacement, thus ensuring that all CpGs of a given locus can be accessed by the large DNMT3A/3L protein complex, and this is particularly relevant since growing oocytes are in meiotic arrest and not dividing [2].

## Materials and methods

### Mouse experimental procedures

All experimental procedures were approved by the Animal Welfare and Ethical Review Body at the Babraham Institute and were performed under licenses issued by the Home Office (UK) in accordance with the Animals (Scientific Procedures) Act 1986.

### RNA-Seq library preparation

Oocytes were collected from 5–20-day-old C57BL/6Babr mice and RNA was extracted using TRIsure reagent (Bioline) followed by RNA Clean & Concentrator (Zymo Research) with on-column DNAse treatment (RNase-free DNase I, Life Technologies). Ribosomal RNA was depleted from total RNA using Ribo-Zero Magnetic Kit (Human/Mouse/Rat — Low Input, Epicentre). Libraries from GO1 and GO2 were prepared using ScriptSeq v.2 RNA-Seq Library Preparation Kit (Epicentre). To generate sequencing libraries from NGOs and FGOs reverse transcription was performed using SuperScript III (Life Technologies), followed by second DNA strand synthesis using dUTPs instead of dTTPs and DNA polymerase I (NEB); libraries were constructed using the NEBNext DNA Library Prep Master Mix Set for Illumina (NEB), including dUTP excision step by USER Enzyme (NEB) before PCR.

### Library sequencing and mapping

NGO, GO1, GO2 and FGO RNA-Seq libraries were sequenced with 100-bp paired-end reads on an Illumina HiSeq1000. Raw reads were trimmed to remove both poor quality calls and adapters using TrimGalore v.0.2.8 and mapped to the mouse genome (GRCm38 assembly) using TopHat v.2.0.9 (option –g 1). Published RNA-Seq datasets (Table S2 in Additional file 2) were re-mapped using the same approach in conjunction with gene models from Ensembl release 70, except for the Park et al*.* dataset, for which TopHat v.2.0. 9 (options -- color --quals –g 1) was used. Mapping of reads to repetitive elements is described in Additional file 3.

BS-Seq published datasets (Table S2 in Additional file 2) were trimmed using TrimGalore v.0.2.7 with default parameters, aligned to the mouse genome GRCm38 assembly using Bismark v.0.10.1 (options --pbat, --phred33-quals) [48]. CpG methylation calls were extracted from the deduplicated mapping output ignoring the first 4 bp of each read (for post-bisulfite adaptor tagging (PBAT) libraries with 4N adapters) using the Bismark methylation extractor (v0.10.0; --no_overlap --report --ignore 4 --ignore_r2 4 for paired-end mode; --report --ignore 4 for the single-end mode).

### Oocyte transcriptome assembly

Transcriptome was assembled using Cufflinks v.2.1.1 [23, 24] with default parameters (genome-guided Cufflinks) on a single dataset created by remapping NGO, GO1, GO2, and FGO RNA-Seq datasets (no gene model specification and merging using SAMtools v.0.1.18) and as RABT assembly [25] (option –g) on individual NGO, GO1, GO2, and FGO and merged GO1 and GO2 datasets. For the RABT output, threshold FPKM values to filter non-transcribed transcripts were determined as the point of maximum difference between the values of cumulative distributions of FPKM values of transcripts in the annotation and of random size-matched intergenic regions using a custom R script. FPKM values were determined using Cufflinks v.2.1.1 with -G option. Transcripts that did not exceed the threshold FPKM were removed. Annotation from genome-guided Cufflinks and filtered annotations from RABT assembler were merged into a single annotation by Cuffmerge. Potential artefacts in the assembly were detected by visual inspection. Modifications of the annotation GTF file were performed using custom Perl and Java scripts available on request. More details about identification and assessment of the artefacts in the assembly are in Additional file 3.

### Curation of the raw Cufflinks annotation

First, transcripts present in the individual datasets but omitted by Cuffmerge were re-integrated. In some instances, reference transcripts were wrongly assigned FPKM values of 0 by Cufflinks, and re-quantifying the expression of these genes independently led to an increase in almost 2000 predominantly multi-exonic transcripts. In addition, a large number of mono-exonic transcripts (48,232) were found in the raw oocyte annotation, suggesting that some of them could be artefacts; therefore, we applied more stringent criteria for this category. For instance, we removed transcripts without clear directionality information, and transcripts located in introns of multi-exonic genes with the same strand orientation that could correspond to remnants of nascent transcripts. We removed mono-exonic transcripts wrongly defined because of issues with the read aligner TopHat (in which a read can be aligned to multiple positions with the same mapping score). We also observed numerous mono-exonic transcripts of the same directionality grouped in clusters, and these were frequently found 3′ of multi-exonic transcripts. Since these arrays could result from the incomplete annotation of single longer genes or extended multi-exonic transcripts, we merged those transcripts present within a 2-kbp genomic interval of a 3′ end (after having tested multiple size windows and assuming that, theoretically, the number of independent mono-exonic genes on the same strand and on the opposite strand 3′ to a gene should be the same). Finally, since mono-exonic genes can be small, their FPKM values can be relatively high, resulting in artefacts caused by the background noise in RNA-Seq datasets. We therefore re-quantified mono-exonic genes based solely on read count, and removed low-expressed ones based on cutoffs determined using normalised random intergenic regions. By performing these corrections on the raw Cufflinks output, the number of multi-exonic transcripts was increased from 65,334 to 67,112 and the number of mono-exonic transcripts was decreased from 48,232 to 15,827. Of note, the majority of removed transcripts were shorter than 1 kbp, while the additional transcripts recovered were predominantly longer than 5 kbp (Fig. 1a; Figure S1c in Additional file 1). The output of our Cufflinks assembly and curation is presented as an annotation track (.gtf file) in Additional file 5.

### Transcriptome-related bioinformatic analyses

The reference transcriptome used in this study was generated using Cuffmerge (Cufflinks v.2.1.1) by merging Ensembl, UCSC and RefSeq gene models downloaded from UCSC Table Browser as available on 1 October 2014. Genes were defined as in Cuffcompare within Cufflinks v.2.1.1 output. Oocyte gene coordinates were defined as the most upstream start and the most downstream end coordinates from all transcripts per gene. Transcripts were categorised into reference and novel by Cuffcompare, with categories =, c, j, and o marking the transcripts of reference genes and categories i, u, and x novel transcripts.

CGIs and igDMRs were defined as published [7, 9, 49, 50] and lifted over using the UCSC liftover tool into the GRCm38 assembly, removing CGIs on Y chromosome. CGIs were classified as TSS-associated if they overlap the most upstream TSS of a gene ±100 bp, intragenic if they overlap the gene but are not at the TSS, and intergenic without gene overlap. Coordinates for TEs (L1 and L2 LINEs, S2 and S4 SINEs, ERV1s, ERVKs, ERVLs, MaLRs) for the mouse GRCm38 genome build were generated using RepeatMasker. TSSs were classified as CGI-associated if a first base pair of a gene or transcript ±100-bp overlapped a CGI and as TE-associated if a first base pair of a gene or transcript overlapped a TE on the same strand.

Expression of assembled transcripts in published oocyte, embryonic, and differentiated cell datasets (Table S2 in Additional file 2) was quantified using Cufflinks v.2.1.1 (option -G). Expression of genes was determined as a sum of FPKM values of all transcripts per gene. Expression levels in individual embryonic datasets (single cells) were merged per stage taking the total read count in each dataset into consideration. Expression of upstream and reference TSSs at each stage or cell type was estimated in Seqmonk for exons containing upstream or reference TSSs as read count quantification corrected for length and then manually corrected for read count in individual or merged datasets to obtain RPKM values.

To perform hierarchical clustering, only genes with a FPKM value of at least 0.1 in at least one dataset were selected. Log transformed values were mean-centred and clustered based on Pearson’s correlation using the hclust function in R v.3.0.2. All statistical analyses (chi-squared tests) were performed in R v.3.0.2.

### Genome-wide DNA methylation analysis

To define hyper- and hypomethylated domains (HyperD, HypoD), probes were designed over CpGs with data [35], merging 50 consecutive CpGs with step size of ten CpGs. Methylation percentage level was then quantified taking into account only CpGs covered by at least five reads and a minimum of three positions to count a probe. Exported data were then processed using custom Perl scripts (available on request) as shown in Figure S4 in Additional file 1. Overlapping windows with methylation level >75 % and <25 % were merged into HyperDs and HypoDs, respectively, splitting overlapped regions between HyperDs and HypoDs into halves. Then, neighbouring domains of the same status were merged if a gap between them was <2 kbp, or if there was a small domain (<1 kbp) of the opposite status between them. Small domains (<2 kpb) were then removed and, again, neighbouring domains of the same status were merged if a gap between them was <2 kbp.

For correlation with the transcriptome, CGIs, TSSs, and 1-kbp regions (three CpGs with at least three reads) with ≥50 % methylation in NGOs or DNMT3A- or DNMT3L-deficient oocytes were excluded from the domains using a custom Perl script. TSSs excluded from the domain designation were defined as 2-kbp regions downstream of a gene’s most upstream TSS. If a domain was divided into more parts, the information about the parental domain was preserved for adequate correlation with transcription and other features.

Oocyte contigs were defined as genomic regions with three or more reads on the same strand in at least one of the oocyte datasets. Enrichment in ERVK elements and in intermediate levels of methylation (25–50 %) in NGOs or DNMT3A- and DNMT3L-deficient oocytes was quantified by the comparison of numbers of non-transcribed (<25 % overlap by transcripts) HyperDs and HypoDs with >50 % overlap with these features, requiring *p* value <0.0001 in chi-squared test.

A FPKM threshold of 0.5 for gene bodies remaining unmethylated was defined by quantification of the proportion of unmethylated gene bodies from all gene bodies with increasing FPKM values (0–0.1, 0.1–0.2, 0.2–0.3, etc.). Below a FPKM of 0.5, more genes were unmethylated than methylated. CpG density and GC content were quantified using a custom Perl script from GRCm38 genome assembly. All methylation levels were quantified in Seqmonk, using the following parameters: three CpGs with a minimum of three reads depth to count a probe for gene bodies; ten CpGs with a minimum of five reads depth for CGIs; a minimum of five reads depth for individual CpGs. Statistical analyses were performed in R v.3.0.2.

### Generation of *Zac1o* conditional deletion mice

The targeting construct was prepared using homologous recombination in bacteria. We inserted one *loxP* site upstream (2.6 kbp) of the *Zac1o* first exon and one *loxP* downstream together with a neomycin selection cassette flanked by *Frt* sites. The targeting construct was electroporated in C57BL/6J Bruce4 ESCs, and correct integration assessed by Southern blot. Chimeric mice were generated by injecting targeted ESCs into C57BL/6J blastocysts and crossed with female Flpe-Cre mice for excision of the selection cassette. Specific deletion of the *Zac1o* first exon and promoter in oocytes was performed by crossing with *Zp3-Cre* mice. For experiments with allelic information, *Zac1o*-floxed or *Zac1o*-deleted female mice were crossed with *M. castaneus* wild-type males (CAST/EiJ).

### Bisulfite-PCR sequencing and COBRA analysis

Oocytes were collected by mouth pipetting as previously described [11], and lysed at 37 °C for 1 h (SDS 0.5 % final, EDTA 0.5 mM final, phosphate-buffered saline, 10 μg of proteinase K). Bisulfite conversion was performed directly on cell lysates. For tissues, DNA was first purified using phenol-chloroform extraction, 500 ng used for bisulfite conversion, and 50 ng equivalent in each PCR reaction. Bisulfite conversion was performed using a commercial kit according to the manufacturer’s recommendations (Sigma, Imprint DNA modification kit, two-step protocol). PCR was performed using Pfu Turbo Cx Polymerase (Stratagene). Primer sequences are available upon request. Cloning and analysis were performed as described elsewhere [5], with 20–25 clones analysed per genomic region and removal of clones with identical patterns of conversion based on both CpG and non-CpG methylation. For COBRA analysis, DNA methylation of the *Zac1* igDMR was assessed using Taq1 restriction endonuclease.

### Chromatin immunoprecipitation in oocytes

Growing oocytes were collected from 15-dpp females as previously described [11], fixed at room temperature in 4 % formaldehyde for 15 min, washed in phosphate-buffered saline with a final wash in less than 5 μl, snap-frozen and stored at −80 °C before lysis. In total, 2180 *Zac1o*-deleted and 1975 wild-type oocytes were processed. Lysis and immunoprecipitation were performed using the True MicroChIP kit (Diagenode AB-002-0016) with the following modifications. Aliquoted oocytes were lysed using 50 μl total lysis buffer tL1 and incubated on ice for 10 min. Equivalent of 150 μl of ice-cold HBBS buffer was added and all lysates were pooled together in 1.5 ml TPX microtubes (Diagenode). Chromatin shearing was performed using the Bioruptor (Diagenode) with five active cycles (30 s ON, 30 s OFF). Tubes were centrifuged at 14,000 g for 15 min at 4 °C and supernatant collected in a 1.5-ml low-binding tube. Ice-cold complete ChIP buffer tC1 (200 μl) was added, and the total volume was divided in three, equally. H3K36me3 (0.25 μg; Active Motif, 61102), 0.5 μg of H3K4me2 (Abcam, ab32356) and 0.25 μg of IgG (Abcam, ab46540) antibodies were used per immunoprecipitation according to the manufacturers’ protocols, except that DNA purification following removal of cross-links was performed using AMPure XP beads (1.8× ratio, Agencourt). Immuno-precipitated material was separated in two equally, and whole-genome amplification was performed according to the manufacturer’s protocol (WGA4, Sigma-Aldrich, starting from step 6) for nine cycles. We subsequently submitted 1 μl to 15 additional amplification cycles for agarose gel visualisation purposes. The remaining amplified material was purified using AMPure XP beads according to the manufacturer’s recommendations (1.8× ratio, Agencourt), and quantitative PCR performed, with quantification as relative enrichment to IgG and correction for primer efficiency. For *Zp3*, two independent PCR assays were designed for intergenic surrounded regions, two for the promoter region, and three for the gene body; for *Ppia*, this was two intergenic, two promoter, and four intragenic regions; for *Fam164b*, this was two intergenic, two promoter, and three intragenic regions; for the *Zac1/Zac1o* regions, this was two independent assays for intergenic regions, two for the *Zac1o* promoter, three for *Zac1o* intragenic regions, and three for the *Zac1* igDMR. All primer sequences are available upon request.

### Chromatin immunoprecipitation in embryos

ChIP of native chromatin was carried out as described previously [42]. Three ChIP assays were performed using independent chromatin preparations, with anti-H3K4me3 (Diagenode pAb 030-050), anti-H3K9ac (Merck-Millipore 06-942), anti-H3K9me3 (Merck-Millipore 07-442) and anti-H4K20me3 (Merck-Millipore 07-463). Analysis of immunoprecipitated chromatin was done as follows: in the input and antibody-bound fractions for each antiserum used, the parental alleles were differentiated by direct sequencing of the PCR products encompassing a strain-specific single-nucleotide polymorphism in the regions of interest. Input and antibody-bound fractions were quantified by real-time PCR amplification with a SYBR Green mixture (Roche) using a LightCycler® 480II (Roche) instrument. Background precipitation levels were determined by performing mock precipitations with a non-specific IgG antiserum (Sigma C-2288) and were only a fraction of the precipitation levels obtained with specific antisera. Bound/input ratios were calculated and normalised to those for the imprinted *KvDMR*, which we showed to be similar in wild-type and mutant embryos.

### Data availability

The datasets supporting the results of this article are available in the Gene Expression Omnibus repository, under accession number [GEO:GSE70116].

## Additional files

Additional file 1:
**Supplementary Figures S1–S7 with their legends.** (PDF 5482 kb) Additional file 2:
**Supplementary Tables S1–S4.** (PDF 240 kb) Additional file 3:
**Supplementary methods, including more details on the transcriptome assembly process.** (PDF 107 kb) Additional file 4:
**A table with the genomic coordinates of our final definition of HyperDs and HypoDs (GRCm38 assembly).** (XLSX 4255 kb) Additional file 5:
**A gtf file corresponding to the annotation of oocyte transcripts according to our transcriptome assembly (GRCm38 assembly; this version does not include expressed independent repetitive elements).** (GTF 50970 kb)

## Abbreviations

bp: base pair
BS: bisulfite sequencing
CGI: CpG island
ChIP: chromatin immunoprecipitation
CNCI: Coding-Non-Coding Index
CPC: Coding Potential Calculator
DNAme: DNA methylation
DNMT: DNA methyltransferase
dpp: days post-partum
E: embryonic day
ESC: embryonic stem cell
FGO: fully grown oocyte
FPKM: fragments per kilobase of transcript per million mapped reads
GO: growing oocyte
HyperD: hypermethylated domain
HypoD: hypomethylated domain
igDMR: imprinted germline differentially methylated regions
ncRNA: non-coding RNA
NGO: non-growing oocyte
PCR: polymerase chain reaction
PGC: primordial germ cell
RABT: reference annotation-based transcript
RNA-Seq: RNA sequencing
RRBS: reduced representation bisulfite sequencing
TE: transposable element
TSS: transcription start site
UCSC: University of California, Santa Cruz
