# Erratum to: Deep sequencing and de novo assembly of the mouse occyte transcriptome define the contribution of transcription to the DNA methylation landscape

**DOI:** 10.1186/s13059-015-0809-8

**Published:** 2015-12-03

**Authors:** Lena Veselovska, Sebastien A. Smallwood, Heba Saadeh, Kathleen R. Stewart, Felix Krueger, Stéfanie Maupetit-Méhouas, Philippe Arnaud, Shin-ichi Tomizawa, Simon Andrews, Gavin Kelsey

**Affiliations:** Epigenetics Programme, Babraham Institute, Cambridge, UK; Bioinformatics Group, Babraham Institute, Cambridge, UK; GReD, CNRS, INSERM, and Clermont University, Clermont-Ferrand, 63001 France; Department of Histology and Cell Biology, Yokohama City University School of Medicine, Yokohama, Japan; Centre for Trophoblast Research, University of Cambridge, Cambridge, UK

After the publication of this work [1], we noticed that in Figure 2 the legends for panels ‘d’ and ‘e’ were accidentally interchanged. The correct legends are given below:

'd' should be: Venn diagram representing the numbers of upstream TSSs of reference genes identified in our transciptome assembly, in PGCs, early embryos and somatic tissues

'e' should be: Pie charts representing the proportion of TSSs overlapping CGIs, TEs or neither (NA) for reference genes, novel upstream TSSs of reference genes and novel genes. For each category, the proportion of each TE family is displayed as a bar graph

The original article was corrected.
