# Supplementary material for: Deep sequencing and de novo assembly of the mouse oocyte transcriptome define the contribution of transcription to the DNA methylation landscape
Source: Genome Biol. 2015 Sep 25;16:209. doi: 10.1186/s13059-015-0769-z (PMC4582738; doi:10.1186/s13059-015-0769-z)
Supplement: Additional file 1: — Supplementary Figures S1–S7 with their legends. (PDF 5482 kb) [file 13059_2015_769_MOESM1_ESM.pdf]

ADDITIONAL FILE1: Supplementary Figures

Deep sequencing and *de novo* assembly of the mouse oocyte transcriptome define the contribution of transcription to the DNA methylation landscape.

Veselovska L*[1], Smallwood SA*[1], Saadeh H[1,2] , Stewart K[1], Krueger F[2], Maupetit-Méhouas S[3], Arnaud P[3], Tomizawa S-I[4], Andrews S[2], Kelsey G[1,5].

Figure S1. Oocyte RNA-seq datasets and transcriptome assembly.

(a) Fraction of the genome and splice sites covered by at least 5 unique reads in our datasets (individual and merged) and individual published oocyte RNA-seq datasets (see **Additional file 2, Table S2** for the full list of datasets). **(b)** Quantification of threshold FPKM values for filtering non-transcribed transcripts from Cufflinks RABT assembler. The threshold value is visualised as the point of maximum difference (black line) between the cumulative distribution of FPKM values of transcripts in the assembly (blue) and of random size-matched intergenic regions (red). Threshold FPKMs determined using this strategy are displayed in the table below, together with, for each oocyte dataset, the proportion of transcripts exceeding this threshold (expressed and kept in the annotation), and the proportion of intergenic regions exceeding this threshold (not expressed transcripts) representing the percentage of false positives. **(c)** Length distribution of transcripts in the original oocyte annotation (beige), and after our annotation curating (blue). **(d)** Numbers of transcripts and genes in our oocyte Cufflinks annotation compared to Ensembl, RefSeq and UCSC reference annotations. Numbers of genes are as defined in Cuffcompare output.

a

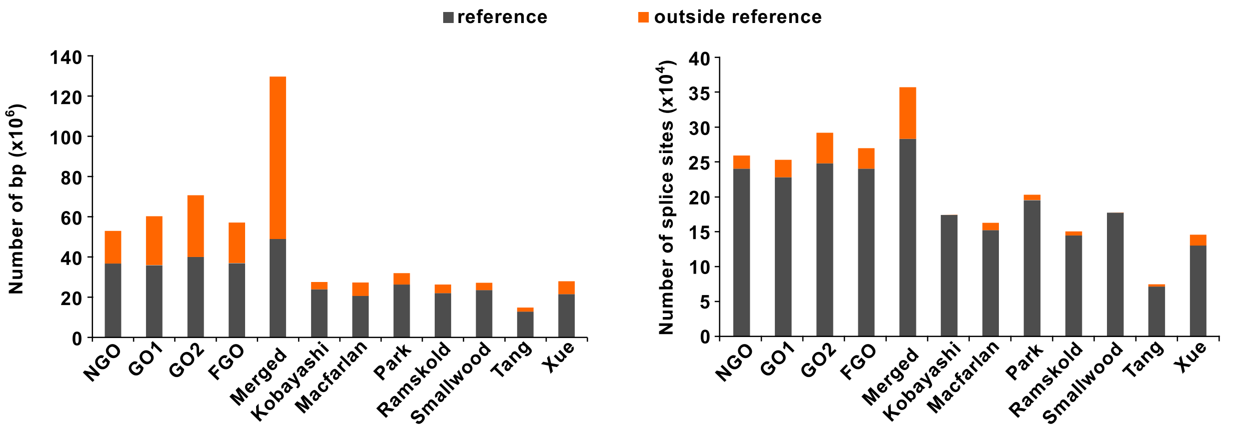

b

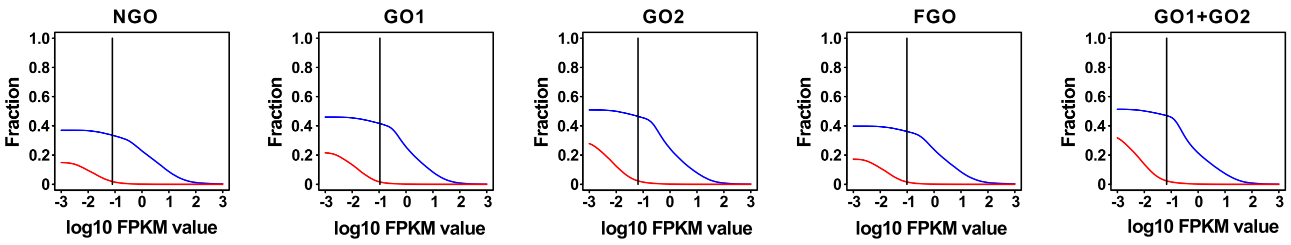

Dataset	log FPKM threshold	FPKM threshold	Proportion of Transcripts kept	Proportion of not expressed transcripts
NGO	-1.1	0.08	33.58%	1.88%
GO1	-0.97	0.11	41.59%	1.60%
GO2	-1.19	0.07	46.48%	2.33%
FGO	-1.01	0.1	36.19%	1.60%
GO1+GO2	-1.17	0.07	47.02%	2.32%

c

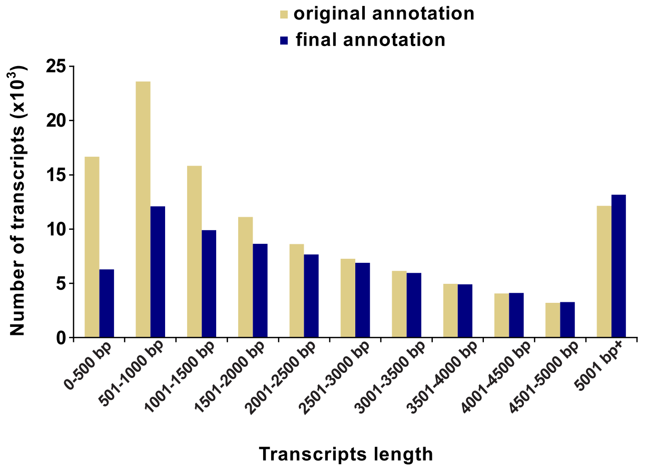

d

	Oocyte Transcriptome	Ensembl	RefSeq	UCSC
Total No. transcripts	82,939	94,084	32,808	58,314
Toal No. genes	39,099	37,319	23,666	30,606
Multi-exonic transcripts	67,112	79,782	28,906	48,713
Mono-exonic transcripts	15,827	14,302	3,902	9,601
Multi-exonic genes	24,104	24,586	20,221	21,784
Mono-exonic genes	14,995	12,733	3,445	8,822

Figure S1

Figure S2. Novel genes and transcripts in the oocyte transcriptome.

(a) Expression of the novel transcripts identified in our assembly in the merged published oocyte RNA-seq datasets. Novel transcripts were divided into five categories according to their expression from the lowest (cat. 1) to the highest (cat. 5) level in our merged RNA-seq datasets. **(b)** Top: validation by RT-PCR of a random selection of novel multi-exonic (L1-L7) and mono-exonic (M1-M7) transcripts. For mono-exonic genes, -/+ indicates controls without reverse transcriptase. Positions of primers relative to the annotation of multi-exonic genes are indicated in the methods. DNA ladders are 100bp (hyperladder IV, Bioline) and 1kbp (hyperladder I, Bioline). Bottom: visualisation of a selection of novel mono-exonic (M4 marked by an asterisk) and multi-exonic genes and their expression in the GO2 RNA-Seq dataset. **(c)** The remaining five clusters with the smallest number of genes from Figure 2c hierarchical clustering.

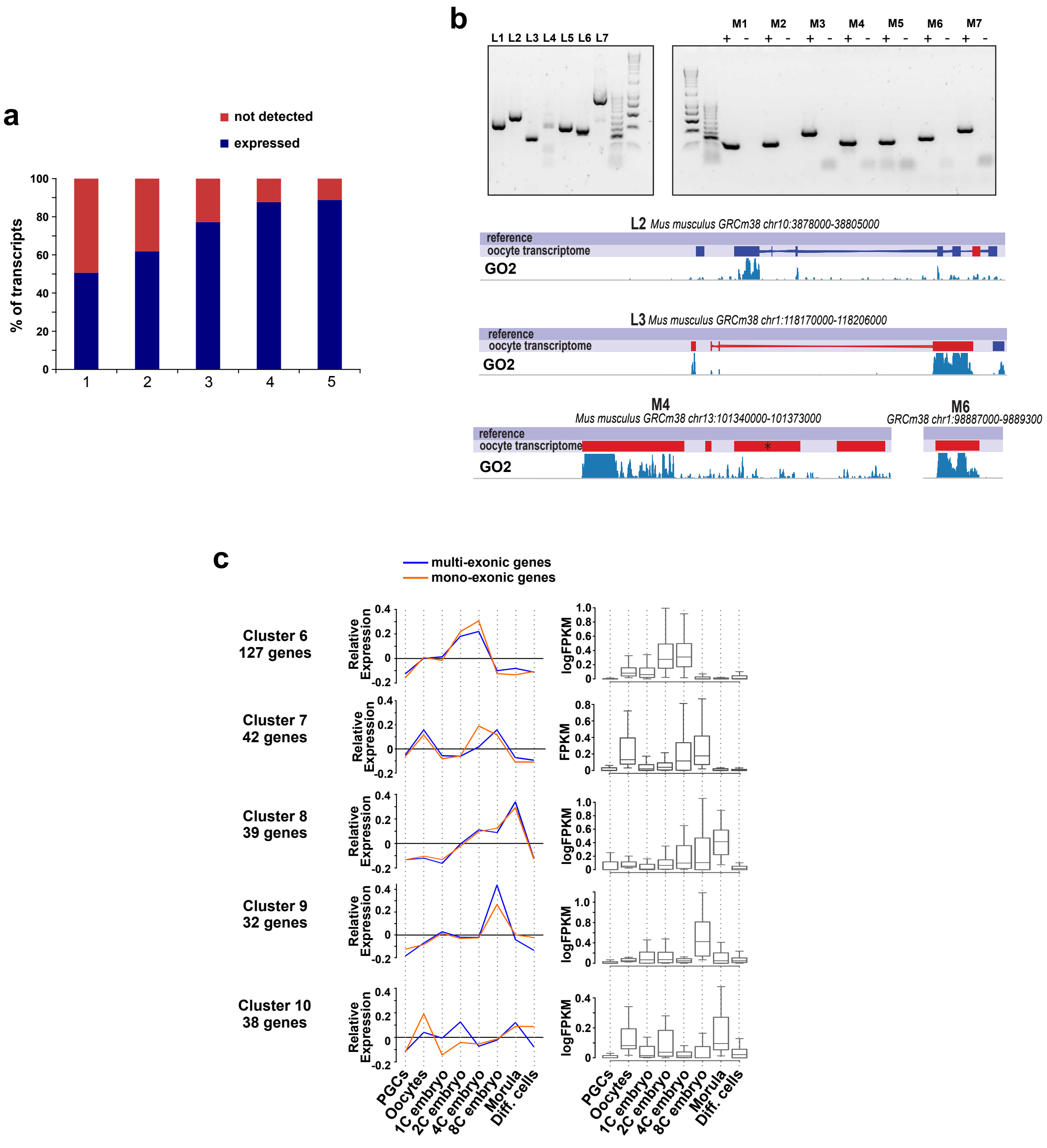
a
not detected
expressed
% of transcripts
b
L1 L2 L3 L4 L5 L6 L7
M1 M2 M3 M4 M5 M6 M7
L2 Mus musculus GRCm38 chr10:3878000-38805000
reference
oocyte transcriptome
GO2
L3 Mus musculus GRCm38 chr1:118170000-118206000
M4 Mus musculus GRCm38 chr13:101340000-101373000
M6 GRCm38 chr1:98887000-9889300
c
multi-exonic genes
mono-exonic genes
Cluster 6
127 genes
Cluster 7
42 genes
Cluster 8
39 genes
Cluster 9
32 genes
Cluster 10
38 genes
Relative Expression
logFPKM
FPKM
PGCs
Oocytes
1C embryo
2C embryo
4C embryo
8C embryo
Morula
Diff. cells

Figure S2

Figure S3. Novel upstream TSSs of reference genes in the oocyte transcriptome.

(a) Expression of novel upstream TSSs of reference genes in merged published oocyte RNA-seq datasets. Novel transcripts were divided into five categories according to their expression from the lowest (cat. 1) to the highest (cat. 5) level in our merged RNA-seq datasets. **(b)** Top: validation of a random selection of novel upstream TSSs of reference genes (T1-T12) by RT-PCR. Primers were designed to span at least one intron. Positions of primers relative to the annotation of multi-exonic genes are indicated in the methods. DNA ladders are 100bp (hyperladder IV, Bioline) and 1kbp (hyperladder I, Bioline). Bottom: visualisation of a selection of novel upstream TSSs and their expression in the GO2 dataset. **(c)** Expression of analysed TE families in our oocyte datasets, assessed by mapping RNA-Seq reads to custom repeat genomes of individual repeat families. **(d)** Expected and observed frequencies of TE families overlapping (+/- 1bp) TSSs on the same strand. Expected frequencies were calculated for the whole genome and for intergenic regions only, as TEs are more frequently found in intergenic than gene regions. Observed frequencies were calculated for (from top in the legend) all reference TSSs (reference all), the most upstream TSSs of reference genes (reference most upstream), TSSs of oocyte genes matching reference genes (oocyte matching reference), all oocyte TSSs (oocyte all), the most upstream TSSs of the oocyte genes (oocyte most upstream), all TSSs of oocyte genes after removal of transcribed independent TEs (oocyte filtered all), and most upstream TSSs of oocyte genes after removal of transcribed independent TEs (oocyte filtered most upstream). Statistically significant enrichment of TEs acting as TSSs after removal of expressed independent TEs compared to the expected frequency by chance in intergenic regions is marked by asterisk ($p<0.0001$, chi-squared test). **(e)** Boxplot representation of the expression levels of genes with a TSS associated with TEs, not associated with TEs and associated specifically with MaLR, ERVK and other TEs, shown with and without outliers. Asterisks mark significant differences (p-value <0.0001, chi-squared test). (**f)** Profiles of TSS activity of reference genes with a novel upstream TSS identified in our transcriptome assembly (1560 in total), in PGCs, early embryos and somatic tissues. Percentage values on the right represent the 4 top categories, and the weight of the lines indicates the proportion of genes with each profile.

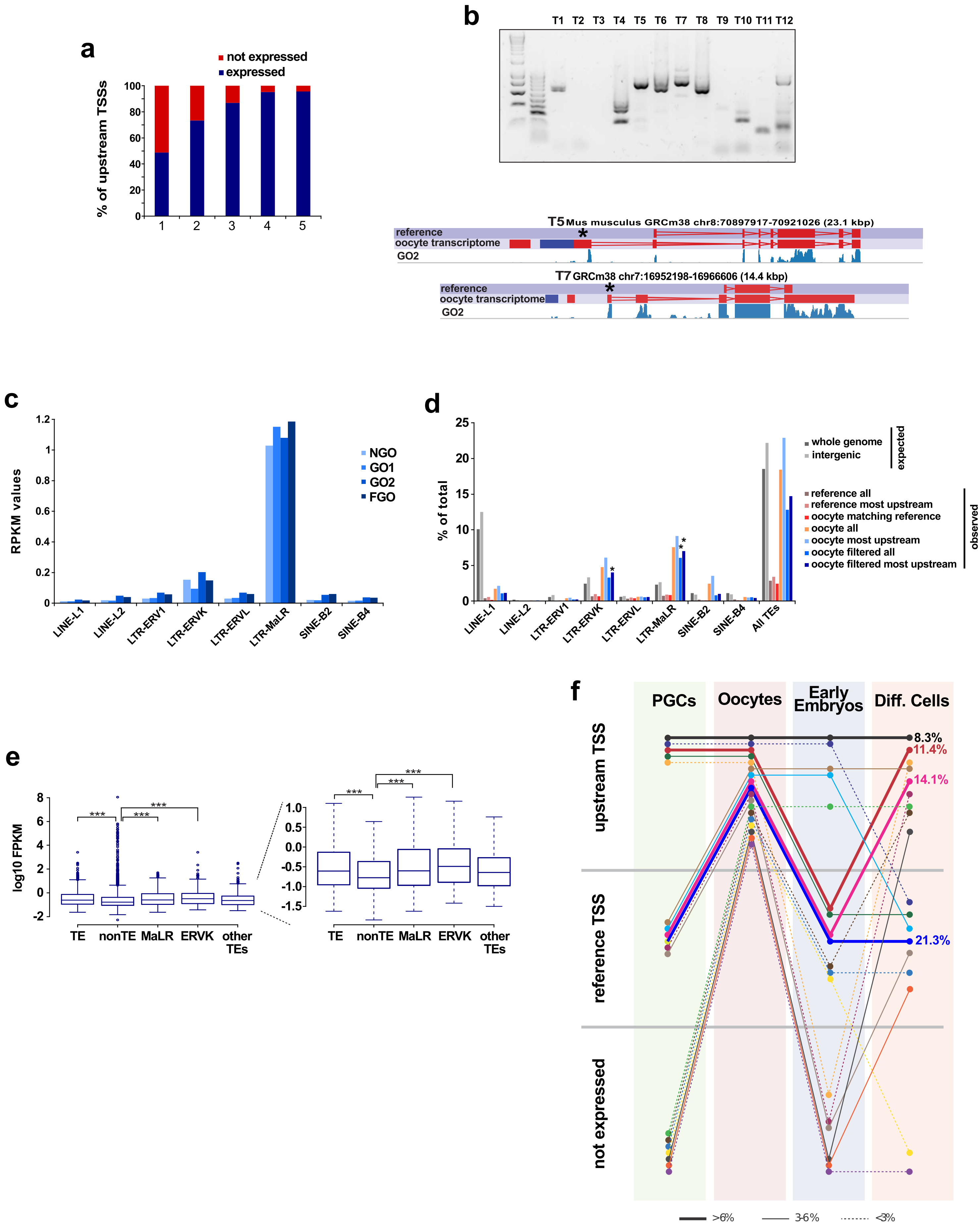
a
not expressed
expressed
% of upstream TSSs
b
T1 T2 T3 T4 T5 T6 T7 T8 T9 T10 T11 T12
T5 Mus musculus GRCm38 chr8:70897917-70921026 (23.1 kbp)
reference
oocyte transcriptome
GO2
T7 GRCm38 chr7:16952198-16966606 (14.4 kbp)
c
RPKM values
NGO
GO1
GO2
FGO
LINE-L1
LINE-L2
LTR-ERV1
LTR-ERVK
LTR-ERVL
LTR-MaLR
SINE-B2
SINE-B4
d
% of total
whole genome
intergenic
expected
reference all
reference most upstream
oocyte matching reference
oocyte all
oocyte most upstream
oocyte filtered all
oocyte filtered most upstream
observed
All TEs
e
log10 FPKM
TE
nonTE
MaLR
ERVK
other TEs
f
PGCs
Oocytes
Early Embryos
Diff. Cells
upstream TSS
reference TSS
not expressed
8.3%
11.4%
14.1%
21.3%
>6%
3-6%
<3%

Figure S3

Figure S4. Definition of HyperDs and HypoDs.

(a) Detailed flowchart of the strategy and parameters used for the definition of HyperDs and HypoDs. The numbers in red indicate the key steps for final definition of domains. **(b)** Length of the domains after each step of domain definition as described in (a) (red numbers).

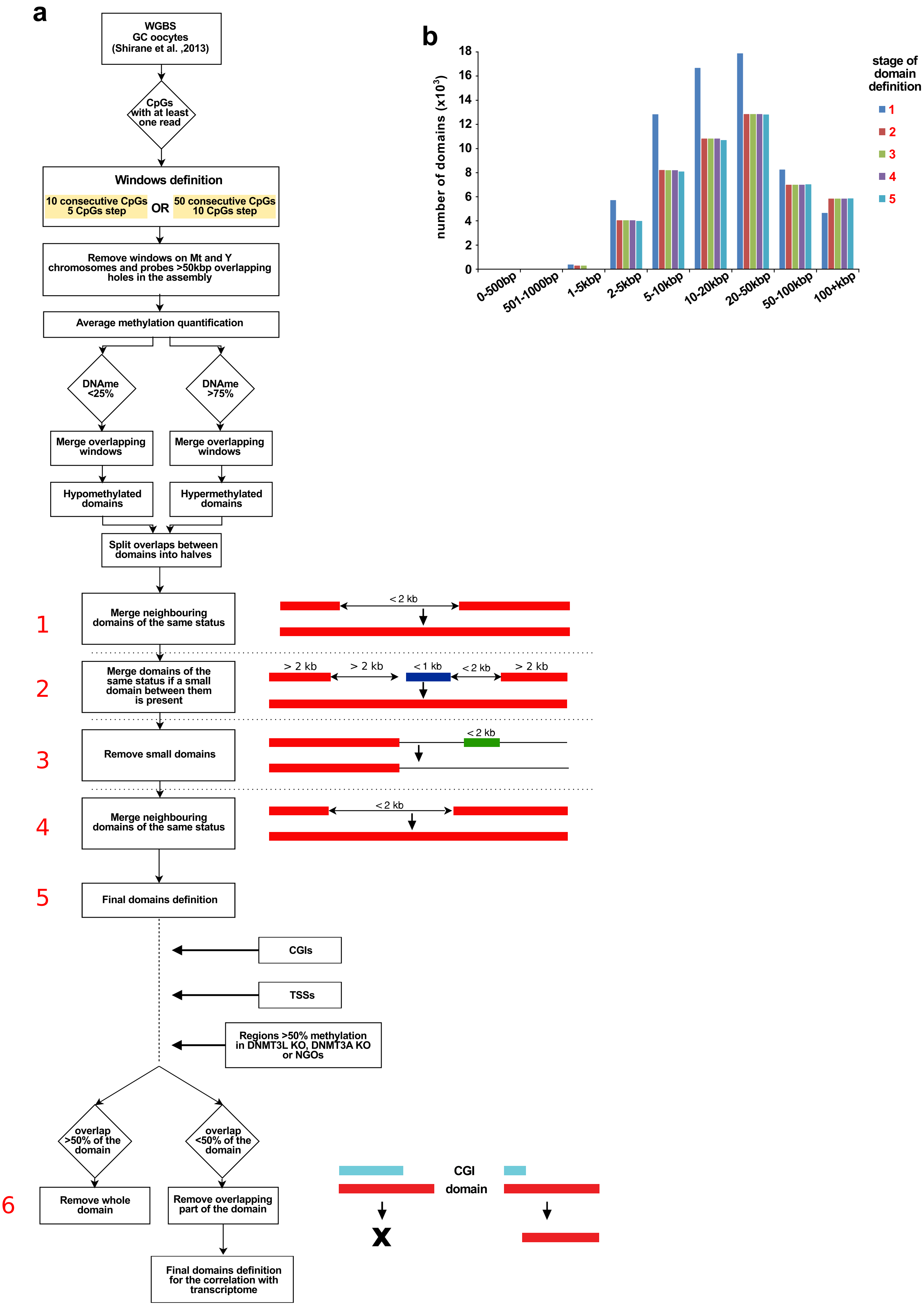
a
WGBS
GC oocytes
(Shirane et al. ,2013)
CpGs with at least one read
Windows definition
10 consecutive CpGs 5 CpGs step
OR
50 consecutive CpGs 10 CpGs step
Remove windows on Mt and Y chromosomes and probes >50kbp overlapping holes in the assembly
Average methylation quantification
DNAme <25%
DNAme >75%
Merge overlapping windows
Merge overlapping windows
Hypomethylated domains
Hypermethylated domains
Split overlaps between domains into halves
1
Merge neighbouring domains of the same status
< 2 kb
2
Merge domains of the same status if a small domain between them is present
> 2 kb
> 2 kb
< 1 kb
< 2 kb
> 2 kb
3
Remove small domains
< 2 kb
4
Merge neighbouring domains of the same status
< 2 kb
5
Final domains definition
CGIs
TSSs
Regions >50% methylation in DNMT3L KO, DNMT3A KO or NGOs
overlap >50% of the domain
overlap <50% of the domain
6
Remove whole domain
Remove overlapping part of the domain
Final domains definition for the correlation with transcriptome
CGI
domain
X
b
number of domains (x10³)
0
2
4
6
8
10
12
14
16
18
0-500bp
501-1000bp
1-5kbp
2-5kbp
5-10kbp
10-20kbp
20-50kbp
50-100kbp
100+kbp
stage of domain definition
1
2
3
4
5

Figure S4

Figure S5. Characteristics of HyperDs and HypoDs and their correlation with transcription.

(a) Distribution of small (<10kbp) and large (>50kbp) HyperDs according their overlap with transcription in oocytes, based on the expressed reference genes (Ref. FPKM>0.001), our transcriptome assembly, our assembly combined with read contigs, our assembly/contig combined with transcribed regions of partial DNAme (>25%) in DNMT KO oocytes and NGOs and overlap with ERVK. **(b)** Proportion of 1kbp windows within HyperDs overlapping with the following features: transcripts of different FPKM values, oocyte contigs, regions within 2 kb downstream of the TTSs of the oocyte transcripts, regions with 25-50% DNA methylation in DNMT3A-deficient, DNMT3L-deficient and oocytes early NGOs (partially methylated regions) and ERVK elements. **(c)** Distribution of small (<10kbp) and large (>50kbp) HypoDs according their overlap with transcription in oocytes, based on the expressed reference genes (Ref. FPKM>0.001), our transcriptome assembly, our assembly excluding genes with FPKM ≤0.5 alone or including also alternative TSSs. **(d)** Proportion of 1kbp windows within HypoDs overlapping with the following features: transcripts of different FPKM values and alternative TSSs. **(e)** Metagene analysis of DNAme level and distribution across gene bodies +/-5kbp as defined in our oocyte assembly, according to their expression levels. Genes were divided into 5 categories of equal numbers based on their FPKM values, from the lowest (0-20%) to the highest (80-100%).

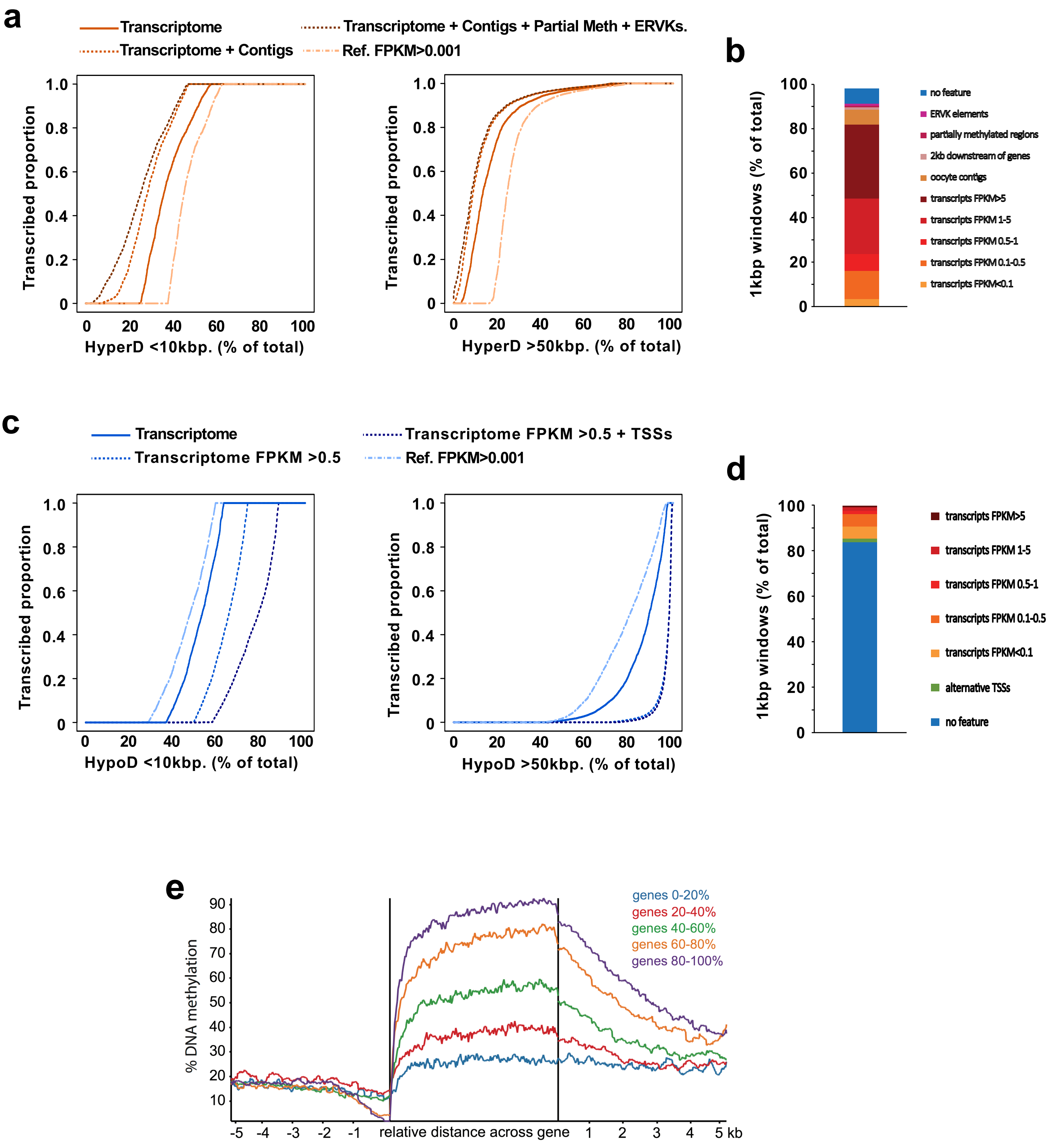
a
Transcriptome
Transcriptome + Contigs
Transcriptome + Contigs + Partial Meth + ERVKs.
Ref. FPKM>0.001
Transcribed proportion
HyperD <10kbp. (% of total)
HyperD >50kbp. (% of total)
b
1kbp windows (% of total)
no feature
ERVK elements
partially methylated regions
2kb downstream of genes
oocyte contigs
transcripts FPKM>5
transcripts FPKM 1-5
transcripts FPKM 0.5-1
transcripts FPKM 0.1-0.5
transcripts FPKM<0.1
c
Transcriptome
Transcriptome FPKM >0.5
Transcriptome FPKM >0.5 + TSSs
Ref. FPKM>0.001
HypoD <10kbp. (% of total)
HypoD >50kbp. (% of total)
d
alternative TSSs
e
% DNA methylation
relative distance across gene
kb
genes 0-20%
genes 20-40%
genes 40-60%
genes 60-80%
genes 80-100%

Figure S5

Figure S6. igDMRs newly identified as intragenic.

Snapshot of RNA-Seq (GO2 and FGO) and DNAme (1kbp windows, BS-Seq, FGO) of the imprinted loci for which igDMRs are newly classified as intragenic according to our transcriptome assembly. Colours of genes and RNA-seq reads represent their strand-specificity (red indicates the alignment to + strand, blue to - strand). For easier interpretation, the RNA-seq reads containing splice sites were visualised as if the read also covered the intron.

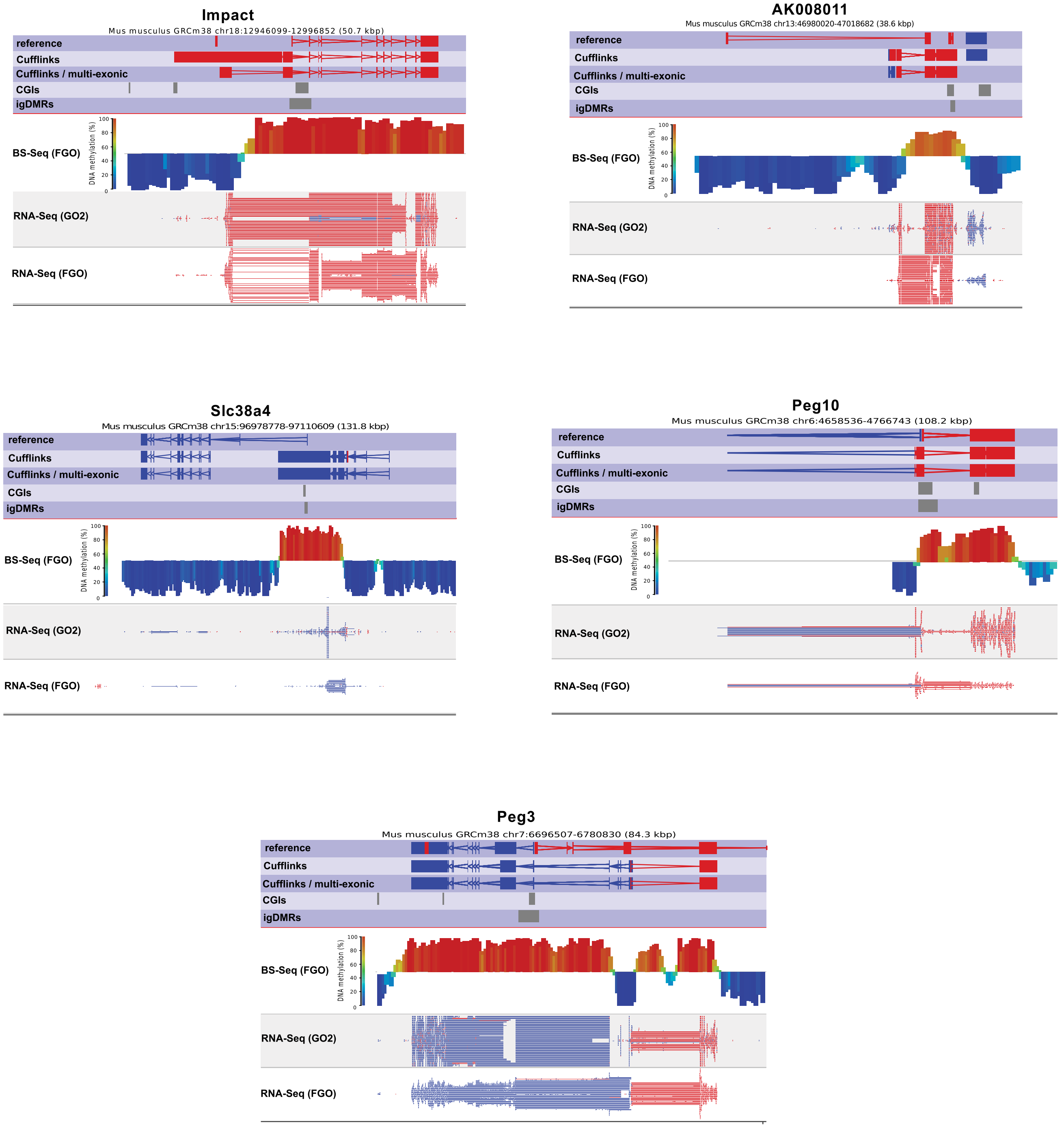
Impact
Mus musculus GRCm38 chr18:12946099-12996852 (50.7 kbp)
reference
Cufflinks
Cufflinks / multi-exonic
CGIs
igDMRs
BS-Seq (FGO)
DNA methylation (%)
RNA-Seq (GO2)
RNA-Seq (FGO)
AK008011
Mus musculus GRCm38 chr13:46980020-47018682 (38.6 kbp)
reference
Cufflinks
Cufflinks / multi-exonic
CGIs
igDMRs
BS-Seq (FGO)
DNA methylation (%)
RNA-Seq (GO2)
RNA-Seq (FGO)
Slc38a4
Mus musculus GRCm38 chr15:96978778-97110609 (131.8 kbp)
reference
Cufflinks
Cufflinks / multi-exonic
CGIs
igDMRs
BS-Seq (FGO)
DNA methylation (%)
RNA-Seq (GO2)
RNA-Seq (FGO)
Peg10
Mus musculus GRCm38 chr6:4658536-4766743 (108.2 kbp)
reference
Cufflinks
Cufflinks / multi-exonic
CGIs
igDMRs
BS-Seq (FGO)
DNA methylation (%)
RNA-Seq (GO2)
RNA-Seq (FGO)
Peg3
Mus musculus GRCm38 chr7:6696507-6780830 (84.3 kbp)
reference
Cufflinks
Cufflinks / multi-exonic
CGIs
igDMRs
BS-Seq (FGO)
DNA methylation (%)
RNA-Seq (GO2)
RNA-Seq (FGO)

Figure S6

Figure S7. Functional test of the involvement of transcription in DNAme establishment.

(a) Scheme of the *Zac1o* conditional KO allele construct and strategy. 2 *loxP* sites were inserted 2.6kbp upstream of the *Zac1o* TSS and just downstream of *Zac1o* exon I, respectively, using homologous recombination in bacteria. The downstream *loxP* site was associated with the Neomycin selection cassette flanked by 3 *Frt* sites that was excised by crossing with Flpe mice. **(b)** Expression of *Zac1o*, *Zac1oAS* and *Ppia* (control) in $Zac1o^{fl/fl}$ and $Zac1o^{fl/fl}$ x Zp3-Cre (*Zac1o-/-*) growing (GO) and fully-grown (FGO) oocytes. (**c**) DNAme at *Zac1* igDMR assessed by COBRA analysis in neonatal (P2) brain (Br) and neonatal (P2) pancreas (Pa) of *Zac1o+/+* and *Zac1o+/-* littermates from two different litters. **(d)** DNAme at *Zac1* igDMR assessed by COBRA analysis in neonatal brain (P2) of *Zac1o+/fl* and *Zac1o+/fl x Sox2-Cre* mice. **(e)** Enrichment of H3K4me3, H3K9me3, H3K9Ac and H3K20me3 at *Zac1* igDMR in *Zac1o+/+* and *Zac1o+/-* (LoM) mice, in neonatal brain. Bound/ input ratios were calculated and normalised to those for the imprinted *KvDMR* (***p<0.001, Wilcoxson test) **(f)** ChIP-qPCR quantification of H3K4me2 and H3K36me3 enrichment in growing oocytes (dpp15) at *Zp3* (highly expressed), *Ppia* (highly expressed) and *Fam164b* (non-expressed) loci, in intergenic, promoter and intragenic regions, for *Zac1o+/+* and *Zac1o-/-* (LoM) mice (*p<0.05, t-student test).

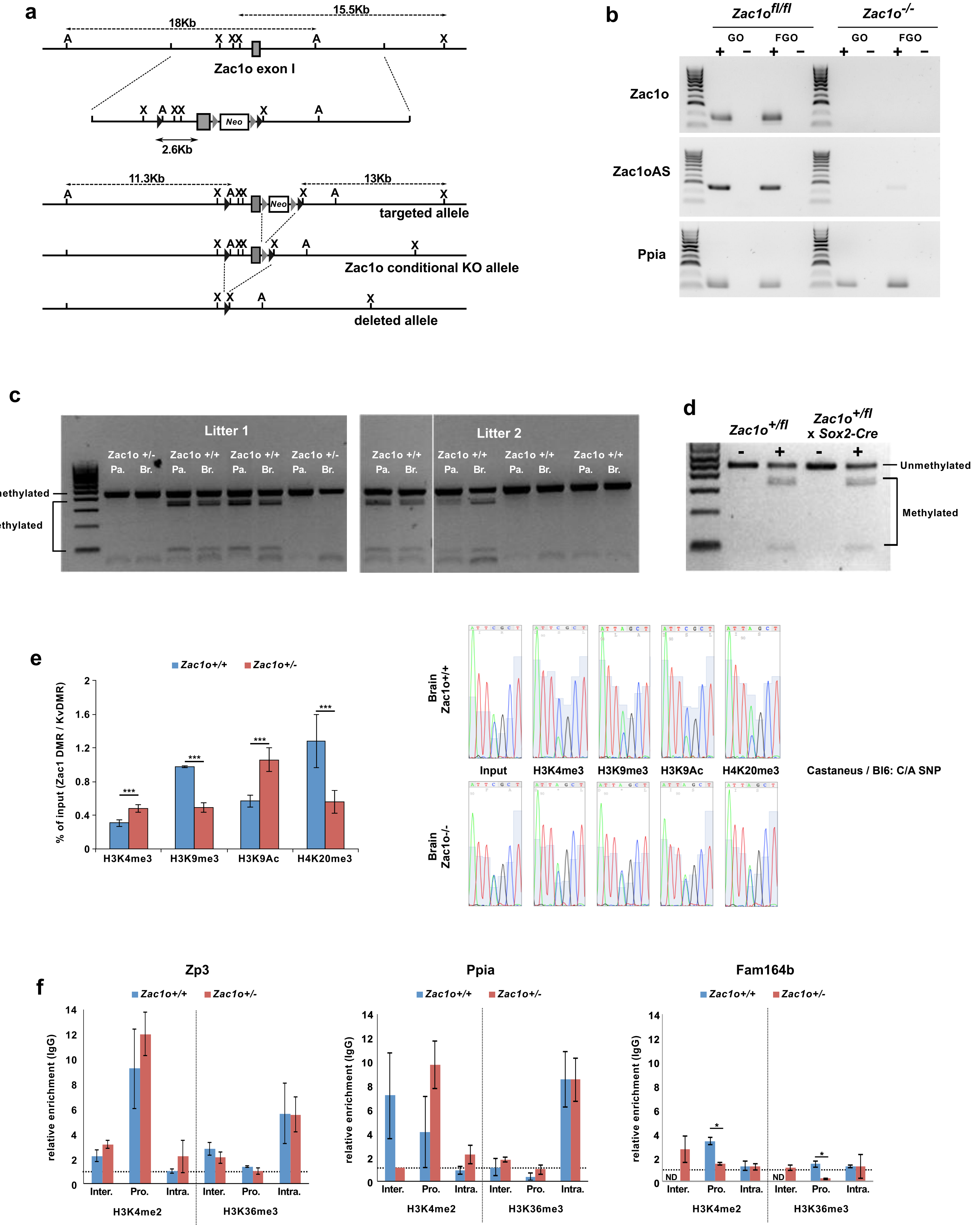
a
15.5Kb
18Kb
Zac1o exon I
Neo
2.6Kb
11.3Kb
13Kb
targeted allele
Zac1o conditional KO allele
deleted allele
b
Zac1o fl/fl
Zac1o -/-
GO
FGO
Zac1o
Zac1oAS
Ppia
c
Litter 1
Litter 2
Unmethylated
Methylated
d
Zac1o +/fl
Zac1o +/fl x Sox2-Cre
e
Zac1o+/+
Zac1o+/-
% of input (Zac1 DMR / KvDMR)
H3K4me3
H3K9me3
H3K9Ac
H4K20me3
Brain Zac1o+/+
Brain Zac1o-/-
Input
Castaneus / Bl6: C/A SNP
f
Zp3
Ppia
Fam164b
relative enrichment (IgG)
Inter.
Pro.
Intra.
H3K4me2
H3K36me3
ND

Figure S7
