# Supplementary material for: Deep sequencing and de novo assembly of the mouse oocyte transcriptome define the contribution of transcription to the DNA methylation landscape
Source: Genome Biol. 2015 Sep 25;16:209. doi: 10.1186/s13059-015-0769-z (PMC4582738; doi:10.1186/s13059-015-0769-z)
Supplement: Additional file 2: — Supplementary Tables S1–S4. (PDF 240 kb) [file 13059_2015_769_MOESM2_ESM.pdf]

ADDITIONAL FILE 2: Supplementary Tables

Deep sequencing and *de novo* assembly of the mouse oocyte transcriptome define the contribution of transcription to the DNA methylation landscape.

Veselovska L*[1], Smallwood SA*[1], Saadeh H[1,2] , Stewart K[1], Krueger F[2], Maupetit-Méhouas S[3], Arnaud P[3], Tomizawa S-I[4], Andrews S[2], Kelsey G[1,5].

Table S1: Comparison of the oocyte transcriptome to the reference annotation using Cuffcompare

Cuffcompare category	Known of novel isoform of	All transcripts	Multiexonic transcripts	Monoexonic transcripts
=	reference genes	21682	21070	612
c	reference genes	1334	1075	259
j	reference genes	27855	27855	0
e		213	0	213
i	novel genes	3950	1621	2329
o	reference genes*	1840	1447	393
p		193	0	193
r		0	0	0
u	novel genes	22830	11615	11215
x	novel genes	2843	2230	613
s		8	8	0
not listed**		191	191	0

*category o contains transcripts with the exonic overlap on the same strand with the reference transcripts. If exonic sequence is shared between two transcripts, they might be different isoforms of the same transcriptional unit.

** oocyte transcripts not listed in the Cuffcompare output share the same splice sites and exons with transcripts listed in the output, but have shorter 5' end of the first exon or 3' end of the last exon.

Table S2: List of RNA-Seq and BS-Seq published dataset used in this study

	Cell type	No of datasets	PMID	GEO accession	PolyA selection/ rRNA depletion	Strand-specificity of the library	Reference
RNA-seq	fully grown oocytes	2	22242016	DRR001701, DRR001702	polyA selected	no	Kobayashi et al., 2012
	fully grown oocytes	3	22722858	SRR385625, SRR385626, SRR385627	polyA selected	no	Macfarlan et al., 2012
	fully grown oocytes	6	24352427	DRR013128, DRR013129, DRR013130, DRR013131, DRR013132, DRR013133	rRNA depleted	yes	Park et al., 2013
	fully grown oocytes	3	22820318	SRR522060, SRR522061, DRR522062	polyA selected	no	Ramskold et al., 2012
	growing and fully grown oocytes	2	21706000	ERR034795, ERR034797	polyA selected	no	Smallwood et al., 2011
	fully grown oocytes	2	21731673	SRR057540, SRR057541	polyA selected	no	Tang et al., 2011
	fully grown oocytes	2	23892778	SRR689233, SRR689234	polyA selected	no	Xue et al., 2013
	fully grown oocytes	2	23892778	SRR689233, SRR689234	polyA selected	no	Xue et al., 2013
	E13.5 and E16.5 female PGCs	2	23219530	ERR192342, ERR192343	polyA selected	no	Seisenberger et al., 2012
	zygote	4	24408435	GSE45719	polyA selected	no	Deng et al., 2014
	zygote	5	24352427	DRR013134, DRR013135, DRR013136, DRR013137, DRR013138	rRNA depleted	yes	Park et al., 2013
	zygote	3	23892778	SRR689235, SRR689236, SRR689237	polyA selected	no	Xue et al., 2013
	2C embryo	4	24408435	GSE45719	polyA selected	no	Deng et al., 2014
	2C embryo	3	22722858	SRR385622, SRR385623, SRR385624	polyA selected	no	Macfarlan et al., 2014
	2C embryo	5	24352427	DRR013139, DRR013140, DRR013141, DRR013142, DRR013143	rRNA depleted	yes	Park et al., 2013
	2C embryo	3	23892778	SRR689238, SRR689239, SRR689240	polyA selected	no	Xue et al., 2013
	4C embryo	4	24408435	GSE45719	polyA selected	no	Deng et al., 2014
	4C embryo	5	24352427	DRR013144, DRR013145, DRR013146, DRR013147, DRR013148	rRNA depleted	yes	Park et al., 2013
	4C embryo	3	23892778	SRR689241, SRR689242, SRR689243	polyA selected	no	Xue et al., 2013
	8C embryo	4	24408435	GSE45719	polyA selected	no	Deng et al., 2014
	8C embryo	3	23892778	SRR689244, SRR689245, SRR689246	polyA selected	no	Xue et al., 2013
	morula	4	24408435	GSE45719	polyA selected	no	Deng et al., 2014
	morula	3	23892778	SRR689247, SRR689248, SRR689249	polyA selected	no	Xue et al., 2013
	brown adipose tissue	2	22989292	SRR496219, SRR496220	n/a	yes	Stamatoyannopoulos et al., 2012
	bone marrow	2	22989292	SRR496247, SRR496248	n/a	yes	Stamatoyannopoulos et al., 2012
	cerebellum	2	22989292	SRR496231, SRR496232	n/a	yes	Stamatoyannopoulos et al., 2012
	cortex	2	22989292	SRR496229, SRR496230	n/a	yes	Stamatoyannopoulos et al., 2012
	heart	2	22989292	SRR496227, SRR496228	n/a	yes	Stamatoyannopoulos et al., 2012
	kidney	2	22989292	SRR496225, SRR496226	n/a	yes	Stamatoyannopoulos et al., 2012
	liver	2	22989292	SRR496235, SRR496236	n/a	yes	Stamatoyannopoulos et al., 2012
	lung	2	22989292	SRR496233, SRR496234	n/a	yes	Stamatoyannopoulos et al., 2012
	spleen	2	22989292	SRR496253, SRR496254	n/a	yes	Stamatoyannopoulos et al., 2012
	embryonic stem cells	2	22989292	SRR496249, SRR496250	n/a	yes	Stamatoyannopoulos et al., 2012
	mouse embryonic fibroblasts	2	22989292	SRR496251, SRR496252	n/a	yes	Stamatoyannopoulos et al., 2012
BS-seq	fully grown oocytes	7	23637617	DRR002141, DRR003599, DRR003600, DRR003601, DRR003602, DRR003603, DRR003604	n/a	n/a	Shirane et al., 2013
	non-growing oocytes	5	23637617	DRR002157, DRR002158, DRR002159, DRR002160, DRR002161	n/a	n/a	Shirane et al., 2013
	DNMT3A-deficient oocytes	3	23637617	DRR002145, DRR002146, DRR002147	n/a	n/a	Shirane et al., 2013
	DNMT3L-deficient oocytes	1	23637617	DRR002151	n/a	n/a	Shirane et al., 2013
	sperm	17	22242016	DRR001664-DRR001679	n/a	n/a	Kobayashi et al., 2012

Table S3: List of expressed genes that escape DNAme targeting in oocytes

ID	Gene Name
Cd59b	CD59b antigen
D0H4S114	DNA segment, human D4S114
Epha7	Eph receptor A7
Isl1	ISL1 transcription factor, LIM/homeodomain
Pdlim3	PDZ and LIM domain 3
Rasa3	RAS p21 protein activator 3
1110007C09Rik	RIKEN cDNA 1110007C09 gene
Fam165b	RIKEN cDNA 1190017O12 gene
Rwdd4a	RWD domain containing 4A
Sox13	SRY-box containing gene 13
Ankmy2	ankyrin repeat and MYND domain containing 2
Asns	asparagine synthetase
Bzw2	basic leucine zipper and W2 domains 2
Fgl1	fibrinogen-like protein 1
Fgf16	fibroblast growth factor 16
Fstl1	follistatin-like 1
Foxo6	forkhead box O6
Fut10	fucosyltransferase 10
H6pd	hexose-6-phosphate dehydrogenase (glucose 1-dehydrogenase)
Hmgn3	high mobility group nucleosomal binding domain 3
Hadh	hydroxyacyl-Coenzyme A dehydrogenase
Krt79	keratin 79
Mfap3l	microfibrillar-associated protein 3-like
Nudt15	nudix (nucleoside diphosphate linked moiety X)-type motif 15
Pax6	paired box gene 6
Gm5595	predicted gene 5595; predicted gene 2381
Shisa6	predicted gene 879
Tpst1	protein-tyrosine sulfotransferase 1
Styxl1	serine/threonine/tyrosine interacting-like 1
Rap2c	similar to RAP2C, member of RAS oncogene family; RAP2C, member of RAS oncogene family
Slit3	slit homolog 3 (Drosophila)
Sohlh2	spermatogenesis and oogenesis specific basic helix-loop-helix 2
Stc2	stanniocalcin 2
Syce1	synaptonemal complex central element protein 1
Tes	testis derived transcript
Tcf4	transcription factor 4
Tmem56	transmembrane protein 56
Ttyh2	tweety homolog 2 (Drosophila); similar to tweety homolog 2
Vps36	vacuolar protein sorting 36 (yeast)
Zbtb38	zinc finger and BTB domain containing 38
Zfp219	zinc finger protein 219

Table S4: List of primer sequences and locations used for for RT-PCR validation

Name*	Forward primer sequence 5'->3'	Reverse primer sequence 5'->3'	Chr	5' end forward primer	5' end reverse primer	Exon number forward primer**	Exon number reverse primer**
T1	ACTGAAAGTGGTGCCATCCC	TCCTGCTCGTCCTTTGTGAT	10	4353241	4253901	1	5
T2	TCGAGGTCTTAGGAGTGGGA	AGCCAGGGTGACATTCATCA	1	136466406	136471456	1	4
T3	GCTGAGGAAATAAGGATGGGG	TGGTGCAGCTAGTGTCTCAT	1	136467589	136471497	1	4
T4	GGCGGCTCCCAACAATTATT	GCAAACAGGTCAGCAGAGAG	2	118554779	118546901	1	3
T5	AGGAGTTGGGAGGAAGAGGA	CGGGAACACATAGCAACAGG	8	70902292	70913172	1	5
T6	CTCAAACACAGCTGTCCACC	TCGTCACACCTGCACAAAAG	16	13769162	13715551	1	6
T7	GAGCAACCTGGTAAGCAGTG	GGAGGCTGGTTTGAATTCGG	7	16955576	16960649	1	3
T8	AGAGGGAATGCCTGTGGATC	CGGGGTGTTTCTTCTTCGTT	13	95546471	95514039	1	4
T9	GAGGTCTTAGGATCCCGTGG	GGGGCGGTTTATGAATACCC	13	93285074	93300740	1	3
T10	GCTACTGTGCCGAATGTGAG	CAGTGGTAGGCAGGTGACAG	7	17685539	17714810	1	3
T11	AGCCTTGCCTGGAGATGAAT	AAGTGAAGCTGGGCAGACA	4	11734374	11767778	1	2
T12	CAGATGACCCTGGCCAGTAG	GTTGAACTGCTGCTAACCGT	4	48506897	48468813	1	4
L1	AGAAGAATCCCACCAACCGT	TGAATTATTCCAGGGGCTCAG	10	31657098	31646408	1	2
L2	TGCTGACTTCTCCCACACAT	TCAACCCTGATAGTGGCTCC	10	38804243	38785926	1	4
L3	TGACATTCATGGATGTGGCT	TGTCACTCGAGTAGAAATTCTGT	1	117605633	117625513	1	2
L4	TCATCCCAACCAGCTCTTCC	AGAGTGTTGGCCTTCTCCC	5	62006230	62013483	1	2
L5	TCACTGGACCAATCAGCCTT	CAGGCATGTTGGATCTGTGC	1	4170278	4147832	1	4
L6	TTCCTCTGCCATGCCATACT	CCCAGAGTTTCCCCTGTCAT	1	18335922	18339445	1	2
L7	GTCTGTTGAGCTGGTTGTCC	GAGTCTCTGGTGTGTGGGAA	13	99826281	99761604	1	4
M1	CGGTGCCAGCATAATGTTCA	GGACAGAGCCCTCATCAGAA	1	5438579	5438363	n/a	n/a
M2	AGCACCTTCTTCAGCCATCT	TCTGAGAAGCTGCAAGTGGA	1	21330669	21330428	n/a	n/a
M3	GACAGGAGAGAAACAGCCCT	CAGTCCGTACCCACATCGTA	1	37542544	37542928	n/a	n/a
M4	AGCCAGAATCCTTCAGTGCT	AGGAGATGGAGGGAGAGGTT	9	98888570	98888823	n/a	n/a
M5	GTCAGCAAAGGGTCACATGG	CATCCCAGAGGTTCCAGAGG	5	137930578	137930841	n/a	n/a
M6	AAGACCCTACGGCCTAATGG	TTTGTGCCGTTTGAGGTAGC	13	101357520	101357820	n/a	n/a
M7	TGAGGCATGGCGATCTTTTG	GGTGGGCTAGAACAACCTCT	13	101345938	101346354	n/a	n/a

** Ts are transcripts starting at upstream TSSs of reference genes, Ls novel multiexonic genes and Ms novel monoexonic genes*

*** Numbers indicate the order of the exons within transcript structure from 5' end to 3' end*
