# Supplementary material for: Deep sequencing and de novo assembly of the mouse oocyte transcriptome define the contribution of transcription to the DNA methylation landscape
Source: Genome Biol. 2015 Sep 25;16:209. doi: 10.1186/s13059-015-0769-z (PMC4582738; doi:10.1186/s13059-015-0769-z)
Supplement: Additional file 3: — Supplementary methods, including more details on the transcriptome assembly process. (PDF 107 kb) [file 13059_2015_769_MOESM3_ESM.pdf]

ADDITIONAL FILE3: Supplementary Methods

Deep sequencing and *de novo* assembly of the mouse oocyte transcriptome define the contribution of transcription to the DNA methylation landscape.

Veselovska L*[1], Smallwood SA*[1], Saadeh H[1,2] , Stewart K[1], Krueger F[2], Maupetit-Méhouas S[3], Arnaud P[3], Tomizawa S-I[4], Andrews S[2], Kelsey G[1,5].

Mapping of reads to repetitive elements

To analyse the expression of repetitive elements, RNA-seq reads were aligned to the custom repeat genomes of individual repeat families using Bowtie v1.1.0 with default parameters. To construct repeat genomes, all instances of a given repeat family from the repeat annotation file were extracted into separate BED files. The sequence of each instance from a given repeat family was extracted from the GRCm38 genome using a custom script and concatenated into a single long genome sequence for each family. Individual instances of repeats were separated by NNNNN to prevent sequences from mapping over instance boundaries. The repeat genomes were indexed with Bowtie-build v1.1.0.

Identification, assessment and removal of artefacts in the transcriptome assembly

Transcripts of genes omitted by Cuffmerge were identified using Seqmonk (http://www.bioinformatics.babraham.ac.uk/projects/) as transcripts in the filtered RABT assembly output annotation (input for Cuffmerge) missing in the Cuffmerge output annotation, and added by Unix function cat. Transcripts of genes with incorrectly determined FPKM value as 0 were detected by selecting genes with 0 FPKM value (estimated by Cufflinks) in all input datasets and re-quantifying their FPKM values using Cufflinks v2.1.1 (-G option). Transcripts that exceeded the previously defined FPKM threshold in each dataset were merged with the oocyte annotation by Cuffmerge. Duplicated transcripts were defined as transcripts with the same exonic locations. Transcripts without strand determination and mono-exonic genes within introns of the same strand multi-exonic genes were identified and removed with custom Java scripts.

To identify mono-exonic genes with ≥50% of reads mapping to multiple locations, reads overlapping mono-exonic genes were split into singletons (no paired read or paired read outside mono-exonic gene annotation), improper pairs (individual reads are on different chromosomes), proper pairs with unique mapping and proper pairs with multiple mapping. To achieve this, the GTF file with mono-exonic genes was converted to BED format using Bedops (Neph, et al., 2012) v2.2.0 and BAM file for each dataset was filtered to contain only reads within the BED annotation using SAMtools v0.1.18. Filtered BAM files were sorted using SAMtools v0.1.18. A custom Perl script was used to generate BAM files with singleton reads and improper pairs and write the sequences of reads in proper pairs into fastq files. Fastq files for each dataset were remapped to mouse genome GRCm38 assembly using TopHat v2.0.11 (option -g 2). With this information, a custom Perl script was used to re-classify the lines from the BED-filtered BAM file that were not used to generate singletons and improper pairs BAM files and distinguish between reads mapping to unique or multiple locations, creating BAM files for proper pairs with unique mapping and proper pairs with multiple mapping. The number of reads on the correct strand in each BAM file (merging all oocyte datasets) overlapping mono-exonic genes was quantified using Seqmonk and a ratio of read count of proper pairs with unique mapping to all paired reads (including improper pairs) was estimated for each mono-exonic genes. Mono-exonic genes with a ratio lower than 0.5 were excluded.

To define a cut-off for the window size within which to merge the same strand mono-exonic genes to 3' ends of other mono-exonic genes or multi-exonic genes, the number of mono-exonic genes within a particular window on the same strand and on the opposite strand was counted, for window sizes between 500 bp and 10 kb. As the number of genes followed at the 3' end by an mono-exonic gene on the same strand and on the opposite strand should be the same, the number of mono- or multi-exonic genes with mono-exonic genes on the opposite strand at their 3' end should be the same as the number mono- or multi-exonic genes with the real independent same strand mono-exonic gene at their 3' end within particular window. The number of mono- or multi-exonic genes that would be extended at their 3' end correctly or incorrectly was estimated for each size window, and assessed whether using a larger window compared to the smaller window would increase the number of correctly extended genes more than the number of incorrectly extended genes, defining a cut-off of 2 kb. All mono-exonic genes within 2 kb were merged to the 3' ends of the same strand mono- or multi-exonic genes using a custom Java script. It should be noted that the threshold of 2 kb at the 3' ends of genes is also applied in the settings of the Cuffcompare program from the Cufflinks package to distinguish between the

incomplete annotation for the alternative 3' and of the gene and an independent mono-exonic gene. Read count threshold to remove mono-exonic genes with low read count was defined by the same approach as for threshold FPKM estimation, using read counts (merging all four RNA-seq datasets together) for mono-exonic transcripts and intergenic controls instead of FPKM values. Mono-exonic genes with read count that did not exceed the threshold were removed.

Additional bioinformatic analyses

Intragenic CGIs were classified as alternative TSS-associated if they overlapped alternative TSS±100 bp. Due to the inaccuracies in the transcriptome assembly caused by preferential distribution of reads to reference transcripts by the RABT assembler, some alternative TSSs in the oocyte transcriptome also present in the reference may not be expressed in oocytes. Therefore, we classified these CGIs primarily as intragenic. Intragenic CGIs were classified as in close proximity to a TSS if they overlap a region within 2 kb downstream of a TSS, based on the observation that regions up to 2 kb downstream from an active TSS can be unmethylated. In case of methylated CGIs, if a CGI was both the most upstream TSS-associated relative to one gene and intragenic relative to other gene, it was classified as intragenic. Expected frequencies of TSSs overlapping TEs were determined according to the genome or intergenic regions occupied by individual TE families, excluding regions in the genome without determined sequence. Novel transcripts, genes and upstream TSSs of known genes were considered expressed in published oocyte datasets and other cell types if their expression level exceeded the threshold defined the same way as for our datasets comparing FPKM distribution of transcripts and random intergenic regions, as described in the main methods. Information about domains within the homology regions of novel potentially protein-coding genes to known protein-coding genes was extracted from the Uniprot database as available on 3/10/2014 with CPC output containing full blastx hits using a custom Perl script.

Validation of novel oocytes transcripts and TSS.

RT-PCR to confirm the presence of novel transcripts or transcript isoforms was performed using oocytes from 15 dpp mice. cDNA synthesis was performed using SuperScript III Reverse Transcriptase (Life Technologies). For monoexonic genes, an RT- reaction was performed as a control. PCR primers were designed using Primer3 v4.0.0). Primers for novel multiexonic genes and novel upstream TSSs of reference genes were

designed to span at least one intron. Sequences of the primers, genomic coordinates of the amplicons and information about exons in which the sequences of the primers are located are listed in Additional File2, Table S4. Touchdown PCR was performed with 1 U HotStarTaq DNA polymerase (Qiagen).
